# Injectable and Assembled Calcium Sulfate/Magnesium Silicate 3D Scaffold Promotes Bone Repair by In Situ Osteoinduction

**DOI:** 10.3390/bioengineering12060599

**Published:** 2025-05-31

**Authors:** Wei Zhu, Tianhao Zhao, Han Wang, Guangli Liu, Yixin Bian, Qi Wang, Wei Xia, Siyi Cai, Xisheng Weng

**Affiliations:** 1 Department of Orthopedics, State Key Laboratory of Complex Severe and Rare Diseases, Peking Union Medical College Hospital, Chinese Academy of Medical Sciences & Peking Union Medical College, Beijing 100730, China; zhuwei9508@163.com (W.Z.); zhaoth18@student.pumc.edu.cn (T.Z.); hannah0928@hotmail.com (H.W.); 13146171903@163.com (G.L.); yxb8408@163.com (Y.B.); wangsq19@student.pumc.edu.cn (Q.W.); 2 State Key Laboratory of Complex Severe and Rare Diseases, Peking Union Medical College Hospital, Chinese Academy of Medical Sciences & Peking Union Medical College, Beijing 100730, China; 3 Centro Médico de Macau do Peking Union Medical College Hospital, Macau 999078, China; 4 Applied Materials Science, Department of Materials Science and Engineering, Uppsala University, 752 37 Uppsala, Sweden; wei.xia@angstrom.uu.se

**Keywords:** bioceramics, scaffold, injectable, osteonecrosis of the femoral head, bone regeneration

## Abstract

(1) Background: Osteonecrosis of the femoral head (ONFH), caused by insufficient blood supply, leads to bone tissue death. Current treatments lack effective bone regeneration materials to reverse disease progression. This study introduces an injectable and self-setting 3D porous bioceramic scaffold (Mg@Ca), combining MgO + SiO_2_ mixtures with α-hemihydrate calcium sulfate, designed to promote bone repair through in situ pore formation and osteoinduction. (2) Methods: In vitro experiments evaluated human bone marrow mesenchymal stem cell (h-BMSC) proliferation, differentiation, and osteogenic marker expression in Mg@Ca medium. Transcriptome sequencing identified bone development-related pathways. In vivo efficacy was assessed in a rabbit model of ONFH to evaluate bone repair. (3) Results: The Mg@Ca scaffold demonstrated excellent biocompatibility and supported h-BMSC proliferation and differentiation, with significant up-regulation of *COL1A1* and *BGLAP*. Transcriptome analysis revealed activation of the PI3K-Akt signaling pathway, critical for osteogenesis. In vivo results confirmed enhanced trabecular density and bone volume compared to controls, indicating effective bone repair and regeneration. (4) Conclusions: The Mg@Ca scaffold offers a promising therapeutic approach for ONFH, providing a minimally invasive solution for bone defect repair while stimulating natural bone regeneration. Its injectable and self-setting properties ensure precise filling of bone defects, making it suitable for clinical applications.

## 1. Introduction

Osteonecrosis of the femoral head (ONFH) develops when a compromised blood supply disrupts bone tissue viability in the femoral head region [1]. This pathological process induces structural deterioration and eventual femoral head collapse, culminating in progressive hip dysfunction and persistent pain [2]. Although comprehensive global epidemiological data remain unavailable, multiple national reports indicate a consistent annual increase in ONFH incidence [1,2,3,4]. China currently reports approximately 8.12 million cumulative cases of non-traumatic ONFH [4]. Current clinical management focuses on decelerating disease progression rather than achieving anatomical reversal, as untreated cases frequently progress to severe functional impairment [5].

For most patients, especially younger ones, hip-preserving treatment is the preferred option for managing ONFH [6,7]. Among these treatments, core decompression is the most commonly used clinical method [8,9]. However, the pathogenesis of ONFH is not fully understood, so core decompression alone is insufficient to prevent the disease from progressing to the stage requiring total hip replacement [9,10,11]. The lack of effective artificial bone repair materials following core decompression remains a significant challenge. Alternative treatments, such as core decompression followed by tamponade, are being explored [12], with various bone fillers like vascularized [13,14] or non-vascularized [15] fibula, β-TCP [16], hydroxyapatite [17], and 3D-printed scaffold [18] under investigation, although they are not yet widely adopted in clinical practice.

Bioceramics represent a category of synthetic bone graft substitutes characterized by multi-component composition and scalable manufacturability [19,20]. Appropriately formulated bioceramics demonstrate mechanical compatibility, biocompatibility, and osteoinductive properties that align with femoral head repair requirements, achieving a dynamic balance between self-degradation and bone tissue regeneration [21,22]. Calcium sulfate, a ceramic-based bone graft material, is frequently combined with water to create injectable pastes for repairing diverse bone defect morphologies [23,24], accounting for its extensive utilization in orthopedic and stomatological applications. Among various hydrates, calcium sulfate hemihydrate (CSH) predominates clinical applications, existing primarily in α and β crystalline forms [23] with the α variant exhibiting superior mechanical strength and lower solubility. This material offers multiple advantages including cost-effectiveness, favorable processability, rapid setting kinetics, appropriate mechanical properties, and biocompatibility [25,26]. Its rapid absorption post implantation and osteogenic stimulation capacity [27] enable in situ formation of porous scaffolds that facilitate vascular infiltration and perivascular mesenchymal tissue development [28,29,30], making it particularly suitable for necrotic femoral head reconstruction post decompression. Molecular investigations reveal calcium sulfate enhances osteoinductive molecule secretion to accelerate osseous regeneration [31,32,33], thereby garnering significant attention in femoral head osteonecrosis research. However, CSH’s suboptimal mechanical performance and mismatched degradation-healing kinetics limit its efficacy in extensive necrosis scenarios [34,35,36], necessitating material optimization. Hybrid scaffolds incorporating oyster shell-derived components with α-CSH have demonstrated enhanced osteogenic activity [37], suggesting composite formulation as a viable strategy. Crystalline magnesium silicate presents complementary attributes including superior mechanical strength and ductility [38], with studies indicating its capacity to modulate ceramic hydration kinetics and prolong scaffold degradation rates to better synchronize with bone healing processes [39]. Furthermore, optimized magnesium silicate incorporation enables sustained Mg^2+^ release that promotes osteoblast recruitment and proliferation while inhibiting bone resorption [40,41,42]. Overall, magnesium-based bioceramic composites are considered ideal for regenerative therapies due to their optimized promotion of bone mineralization and repair [43].

To date, we have developed an injectable assembled 3D porous bioceramic scaffold (Mg@Ca), integrating MgO + SiO_2_ mixtures with calcium sulfate granules. The weight content of MgO and SiO2 in this composite was 20%. The compressive strength of Mg@Ca was approximately 5.0 MPa (indicated by the red box in Figure S1), comparable to that of cancellous bone [44,45]. Owing to the hydration-solidification reaction involving MgO, water, and SiO_2_, Mg@Ca maintains stable compressive strength for bone repair applications. The 20% Mg@Ca composite demonstrates a homogeneous three-dimensional porous structure with optimal porosity and narrow pore size distribution (indicated in Figure S2A–F). This structural uniformity originates from phase transformations during heat treatment. The MgO-SiO_2_ gel system establishes an interconnected porous network during hydration, forming a stable architecture. The high interconnectivity facilitates cellular migration and nutrient transport, while moisture evaporation during thermal treatment further enhances structural stability. This composite has demonstrated in situ formation of three-dimensional porous architecture at the implantation site with enhanced positional stability under physiologically wet conditions [46], theoretically fulfilling the requirements for necrotic femoral head core reconstruction following decompression procedures. The current investigation seeks to systematically elucidate its biological mechanisms and therapeutic efficacy.

## 2. Materials and Methods

### 2.1. Preparation of Mg@Ca Particles

The synthesis of Mg@Ca was performed following a procedure from our previous study [46]. In this study, Mg@Ca particles were prepared via a dry-mixing method as follows: First, 6.4 g of α-calcium sulfate hemihydrate (α-CSH, Honeywell, Stockholm, Sweden), 0.64 g of magnesium oxide (MgO, Sigma-Aldrich, Taufkirchen, Germany), and 0.96 g of fumed silica (SF, Sigma-Aldrich) were thoroughly blended in a Vortex mixer for 5 min. Subsequently, 6.72 mL of deionized water was added to form a homogeneous slurry. The well-mixed slurry was then cast into a custom-designed mold featuring an array of 0.5 mm diameter and 0.5 mm height cavities. After initial curing at room temperature for 4 h, the samples were demolded and subjected to thermal treatment at 190 °C for 0.5–3 h to facilitate the phase transformation from CaSO_4_·2H_2_O to CaSO_4_·1/2H_2_O. The total weight ratio of MgO and fumed silica was precisely maintained at 20% of the formulation, ensuring optimal microstructural configuration and mechanical properties of Mg@Ca. The physical and chemical properties of Mg@Ca were further analyzed by scanning electron microscopy (SEM) and X-ray diffraction (XRD).

### 2.2. Cell Cultures and Differentiation in the Released Medium

Human bone marrow mesenchymal stem cells (h-BMSCs; ScienCell, #7500, Carlsbad, CA, USA) were cultured in Mesenchymal Stem Cell Medium (MSCM, ScienCell, #7501) supplemented with 5% fetal bovine serum, 1% Mesenchymal Stem Cell Growth Supplement (MSCGS), and 1% antibiotics under standard conditions (37 °C, 5% CO_2_, 95% air).

For osteogenic differentiation, biomaterial-conditioned medium was prepared by incubating MSCM with one of three test materials (0.2 g Mg@Ca, 0.5 g β-TCP, or blank control) at a ratio of 10 mL medium per sample for 24 h under the same culture conditions. To eliminate potentially cytotoxic rapidly released ions, the mixture was centrifuged at 1000× *g* for 5 min. The resulting supernatant was discarded, and the remaining material was resuspended in fresh medium for an additional 24 h incubation. The conditioned medium was then sterile-filtered (0.22 μm) prior to cell culture application.

### 2.3. Cell Survival Assay

To evaluate the impact of biomaterials on h-BMSC proliferation, the Cell Counting Kit-8 (CCK8, BOSTER, Wuhan, China) and EdU kit (Beyotime, Shanghai, China) were employed. Initially, h-BMSCs were seeded in 96-well plates at a density of 6 × 10^3^ cells per well and incubated with three different culture media for varying durations (12, 24, 36 h, 5 days, and 7 days). After the designated time points, CCK8 reagent was introduced to each well and allowed to incubate for two hours. The culture medium was refreshed every two days throughout the experiment. Absorbance measurements were taken at 450 nm, and the cell survival rate was determined using the equation:

Cell Survival Rate=[(As−Ab)/(Ac−Ab)]×100%

where *As* represents the absorbance of the experimental well containing cells, biomaterial-released medium, and CCK-8; *Ac* denotes the absorbance of the control well with cells and CCK-8 in basal medium; and *Ab* corresponds to the blank well with only CCK-8 and basal medium (no cells).

For proliferation analysis, h-BMSCs were plated in 24-well plates at 3 × 10^4^ cells/well and maintained in three different culture media for 48 h. The EdU incorporation assay (BeyoClick™ EdU Cell Proliferation Kit with Alexa Fluor 488, C0071S, Beyotime, China) was employed to evaluate cell proliferation. Cells were exposed to 10 μM EdU for 12 h, then fixed with 4% paraformaldehyde for 15 min at room temperature and rinsed three times with PBS. Subsequently, Alexa Fluor 488 conjugation was performed via click chemistry using the kit’s anti-EdU antibody, following the manufacturer’s instructions. Nuclei were counterstained with DAPI. Fluorescence microscopy was used to visualize EdU-positive cells (green) and total nuclei (blue), and proliferation rates were calculated by analyzing the images with ImageJ 1.52 software (NIH, Bethesda, MD, USA).

### 2.4. RNA Extraction and Quantitative Real-Time PCR

h-BMSC cells were used to assess the role of Mg@Ca in osteogenic differentiation in vitro. Based on our previous study, two days of co-culture was appropriate. Three released mediums of biomaterials were described above. Total RNA was extracted from the cultured cells and then underwent reverse transcription with a reverse transcription kit (TransGen Biotech, Beijing, China). Real-time PCR amplification was carried out with SYBR Green detection chemistry (Takara Bio, Shiga, Japan).

### 2.5. Primers Used for Real-Time PCR

Real-time PCR amplification of the cDNA fragment was performed using the primer sequences listed in Table 1.

### 2.6. Transcriptome Sequencing

To investigate the effects of Mg@Ca on osteogenic signaling pathways, h-BMSCs were cultured for 48 h under three experimental conditions. After incubation, cells were harvested by trypsinization (0.25% trypsin-EDTA) and pelleted via centrifugation. The resulting cell suspensions were washed three times with PBS, with supernatants removed after each wash. Total RNA was isolated from each replicate (*n* = 3 per group) using TRIzol reagent (Ambion Life Technologies, Carlsbad, CA, USA) according to the manufacturer’s instructions. RNA integrity and concentration were evaluated with an RNA Nano 6000 Assay Kit on an Agilent Bioanalyzer 2100 system (Agilent Technologies, Santa Clara, CA, USA). Subsequently, RNA-seq libraries were constructed and subjected to high-throughput sequencing on an Illumina HiSeq 2000/2500 platform (Illumina, San Diego, CA, USA). Raw sequencing reads were aligned to the reference genome using TopHat (v2.0.9, Johns Hopkins University, Baltimore, MD, USA), and differential expression analysis was performed with DESeq2 (v1.10.1, Bioconductor). Transcripts exhibiting a false discovery rate (FDR)-adjusted *p*-value < 0.05 were classified as statistically significant.

### 2.7. Animal Study and Surgical Procedure

In our previous study [45], we successfully established a femoral head necrosis model using New Zealand white rabbits (3.0–3.5 kg, supplied by Peking Union Medical College Hospital’s Laboratory Animal Center). The animals were maintained under controlled conditions (25 ± 2 °C, 40–60% humidity) with a 12 h light/dark cycle and ad libitum access to food and water. This study received approval from the Animal Care and Use Committee of Peking Union Medical College Hospital and complied with international guidelines for animal experimentation.

Rabbits were anesthetized by inhalation of isoflurane at 4 L/min. After the lateral surgical area of the right thigh was shaved and sterilized, the animal was fixed on the operating table in the left lateral position. Around the right lateral condyle of the femur, a five cm long incision was made along the long axis of the femur. After the incision of the superficial and deep fascia, the drilled target was 1 cm below its eminence. A five mm diameter Kirschner wire was drilled into the femoral head at 45° with the neck-shaft angle to create a three cm femoral tunnel that reached the epiphysis of the femoral head. Areas of osteonecrosis of the femoral head were created on 24 rabbits. The rabbits were randomly divided into Mg@Ca group (*n* = 12) and negative group (*n* = 12). Mg@Ca was applied to fill the cavity defects in rabbits assigned to the Mg@Ca group. No tissue or materials were added to the defect lumen in the negative group. All procedures strictly adhered to the principle of sterility. Each rabbit received an intramuscular injection of antibiotics 3 days after surgery. The rabbits were allowed to move freely. The rabbits were sacrificed at 4, 8, and 12 weeks after surgery, and the right femurs were harvested and analyzed by micro-CT, Hematoxylin-Eosin staining (HE, Sinopharm, Shanghai, China) and safranin O-fast green staining (Sinopharm).

### 2.8. Statistical Analysis

Results are presented as mean ± standard deviation (SD). Data normality was assessed using the Shapiro–Wilk test, while homogeneity of variance was evaluated with Levene’s test. Normally distributed data with equal variances were analyzed by ANOVA followed by LSD post hoc testing. For non-normally distributed or unequal variance data, the Kruskal–Wallis non-parametric test was employed. All statistical analyses were performed using SPSS software (version 25.0, Chicago, IL, USA).

## 3. Results

### 3.1. Analysis of General Physical and Chemical Properties of Mg@Ca

**Figure 1:**
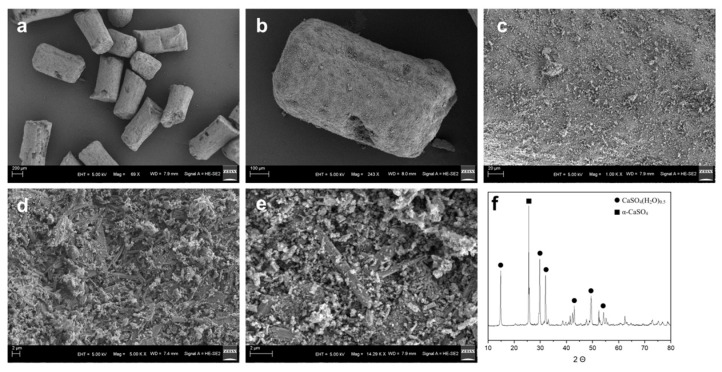
SEM images of Mg@Ca at (**a**) 69×, (**b**) 243×, (**c**) 1000×, (**d**) 5000×, and (**e**) 14,290×. (**f**) XRD of Mg@Ca. All peaks match to CaSO_4_(H_2_O)_0.5_ and α-CaSO_4_.

Mg@Ca was fabricated into cylindrical particles with dimensions of approximately 500 μm in height and 250 μm in radius, enabling adhesion-mediated assembly into a three-dimensional scaffold (Figure 1a,b). SEM observation reveals that the Mg@Ca material possesses a porous and interconnected surface architecture, creating an optimal microenvironment for osteoblast adhesion and migration. The three-dimensional hierarchical pore structure significantly enhances the specific surface area, facilitating cellular pseudopodia extension and mechanical interlocking, thereby improving cell adhesion. Moreover, this porous framework effectively adsorbs bioactive molecules while maintaining efficient nutrient and gas exchange, collectively promoting cellular growth and proliferation.

### 3.2. Assessment of Mg@Ca Scaffold Biocompatibility and Osteoinductive Properties

**Figure 2:**
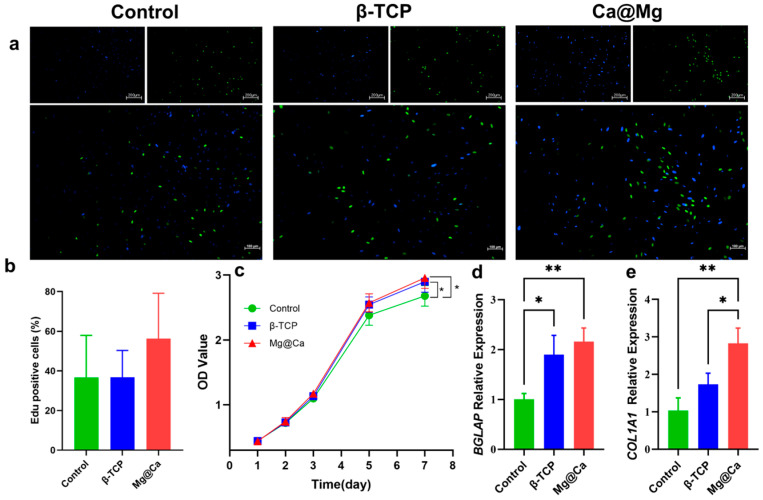
The osteogenic properties of bioceramic scaffolds. (**a**) EdU staining for evaluation of the influences of three groups on the proliferation of h-BMSC. The new generation cells were detected via EdU (green). DAPI stained nuclei in blue. Merged view of EdU (green) and DAPI (blue) showing the overlap. (**b**) Quantitative analysis results of EdU staining using GraphPad Prism 9.0.0 software (La Jolla, CA, USA). (**c**) Quantitative analysis results of CCK-8 staining. (**d**) *BGLAP* and (**e**) *COL1A1* expression of h-BMSCs cultured with blank medium, β-TCP medium, and Mg@Ca medium evaluated by qPCR. Data are presented as mean ± s.d. (*n* = 5). * *p* < 0.05, ** *p* < 0.01 (one-way ANOVA).

The CCK-8 assay was employed to assess h-BMSC viability and quantity on days 1, 2, 3, 5, and 7. All experimental groups demonstrated a consistent upward trend in OD values. Notably, the Mg@Ca group exhibited significantly higher optical density compared to the blank control at day 7 (*p* < 0.05, Figure 2), indicating the scaffold’s excellent biocompatibility and significant growth-promoting properties.

### 3.3. Gene Expression of Osteogenic Genes After Mg@Ca Treatment

Quantitative real-time PCR analysis revealed differential expression of osteogenic genes following Mg@Ca treatment. Consistent with previous findings [46], gene expression differences typically emerged at 48 h, corresponding to the experimental time frame employed. The Mg@Ca group demonstrated significantly elevated *BGLAP* expression compared to controls (*p* < 0.01), though comparable to β-TCP levels. Notably, *COL1A1* expression in the Mg@Ca group surpassed both control (*p* < 0.01) and β-TCP groups (*p* < 0.05) (Figure 2), indicating superior osteoinductive potential of the scaffold material.

### 3.4. Transcriptome Analysis of Mg@Ca

**Figure 3:**
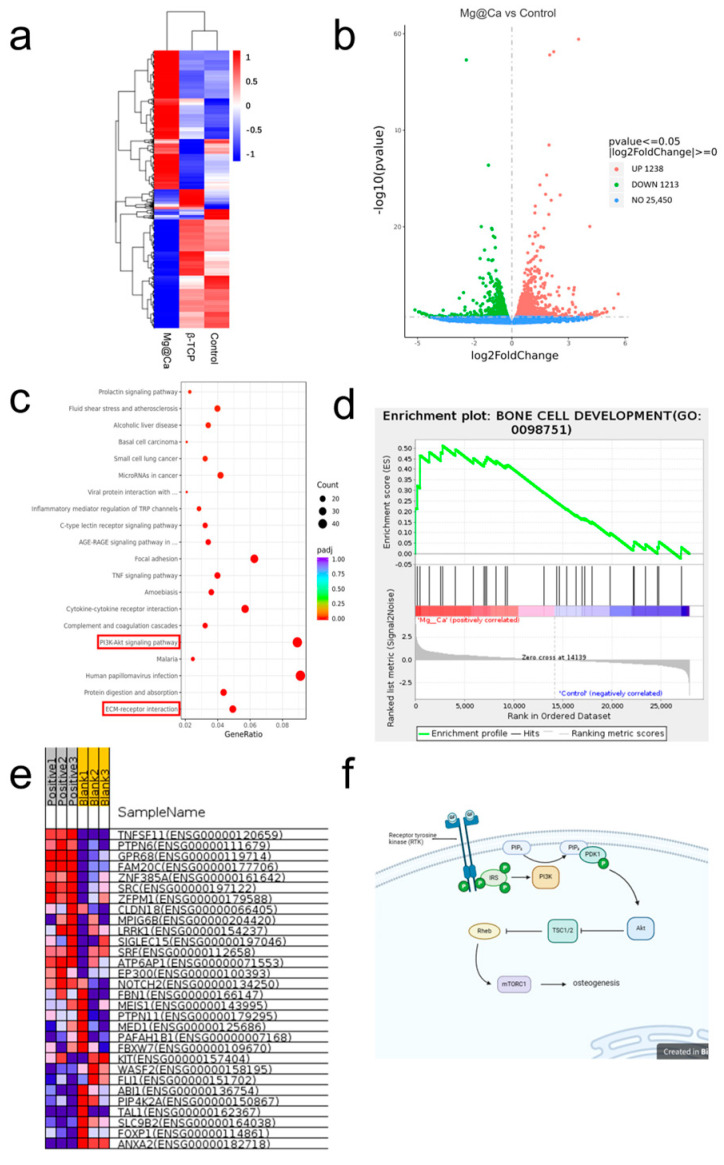
(**a**,**b**) The heatmap and volcano plot of the differentially expressed genes identified by transcriptome sequencing. The up−regulated genes are marked in red, and the down−regulated genes are marked in green. Cutoff: *p*−value < 0.05 and |log2 FC| > 1. (**c**) Top 20 signal pathways enriched by differentially expressed genes using KEGG analysis. The red boxes highlight the PI3K-Akt signaling pathway and ECM-receptor interaction pathway due to their critical roles in osteogenesis. (**d**) GSEA analysis from DisGeNET also indicated enrichment of DEGs in pathways related to BONE CELL DEVELOPMENT. (**e**) DEGs specifically expressed in the Mg@Ca group. Red typically indicates higher gene expression levels, while blue indicates lower gene expression levels across different samples. The identified differentially expressed genes enriched in (**f**) PI3K−Akt signaling pathway on the transcriptome sequencing and IPA 24.0.2 software analysis (created with BioRender.com).

RNA sequencing was performed on h-BMSCs cultured for two days with three release media formulations. By differential gene expression analysis, 2450 differentially expressed genes were found, with 1238 up-regulated and 1213 down-regulated. The top three up-regulated genes with the smallest *p*-value were *NPTX1*, *BDKRB2*, and *PLAU*, and the top three down-regulated genes were *ANKRD1*, *SCUBE3*, and *S1PR1*. GSEA phenotype analysis showed that BONE CELL DEVELOPMENT was activated. KEGG signal transduction analysis showed that the PI3K-Akt signaling pathway and ECM-receptor interaction signaling pathway were highly enriched (shown in Figure 3).

### 3.5. Animal Study

**Figure 4:**
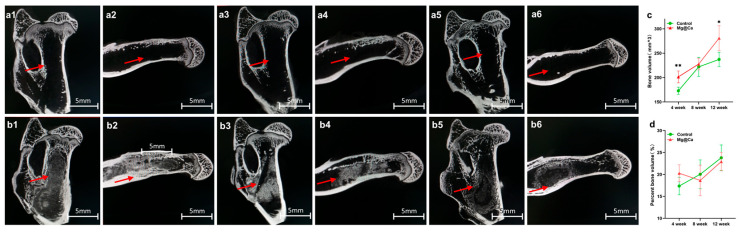
Mg@Ca efficacy in the treatment of rabbit ONFH model: (**a1**–**a6**) Micro-CT scans of the osteonecrosis of the femoral head in the blank group at (**a1**,**a2**) 4 weeks, (**a3**,**a4**) 8 weeks, and (**a5**,**a6**) 12 weeks. The tunnels created by Kirschner (shown by the red arrows) wire remained continuously in the subcapital region. (**b1**–**b6**) Micro-CT scans of the osteonecrosis of the femoral head in the Mg@Ca group at (**b1**,**b2**) 4 weeks, (**b3**,**b4**) 8 weeks, and (**b5**,**b6**) 12 weeks. Mg@Ca within the tunnels was gradually absorbed and replaced by newly formed trabecular bone (shown by the red arrows). (**c**) Quantitative analysis results of bone volume using GraphPad Prism 9.0.0 software. (**d**) Quantitative analysis results of percent bone volume. Data are presented as mean ± s.d. (*n* = 3). * *p* < 0.05, ** *p* < 0.01 (one-way ANOVA).

In vivo ONFH models were established in New Zealand white rabbits using a bore-hole technique, with blank control and Mg@Ca treatments administered at the defect site. Specimens collected at 4−, 8−, and 12−week intervals underwent micro-CT analysis (Figure 4(a1–a6,b1–b6)). Both micro-CT reconstructions and macroscopic observations demonstrated significant repair efficacy in the Mg@Ca-treated group for femoral head condylar cartilage defects. To provide a more comprehensive view, an “micro-CT images” folder is included in the Supplementary Materials, containing more original unformatted images.

**Figure 5:**
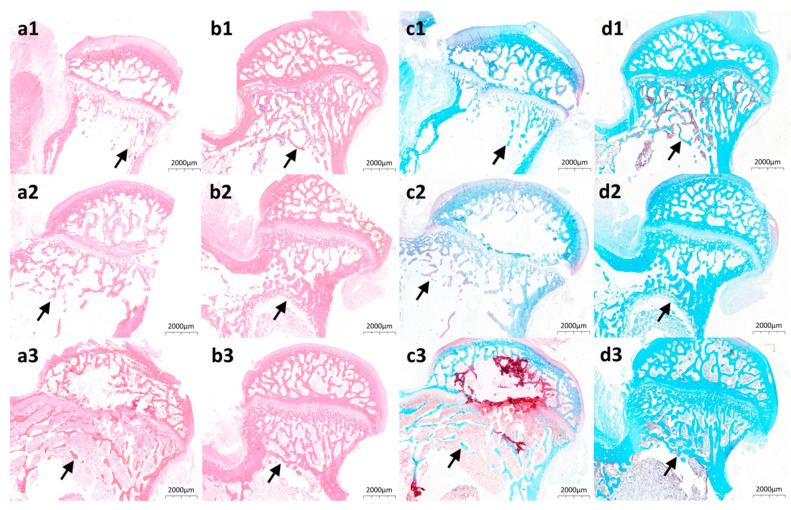
Bone repair promotion by Mg@Ca in rabbit ONFH model: (**a1**–**a3**) HE staining of the osteonecrosis of the femoral head in the blank group at (**a1**) 4 weeks, (**a2**) 8 weeks, and (**a3**) 12 weeks. (**b1**–**b3**) HE staining of the osteonecrosis of the femoral head in the Mg@Ca group at (**b1**) 4 weeks, (**b2**) 8 weeks, and (**b3**) 12 weeks. (**c1**–**c3**) safranin O-fast green staining of the osteonecrosis of the femoral head in the blank group at (**c1**) 4 weeks, (**c2**) 8 weeks, and (**c3**) 12 weeks. (**d1**–**d3**) HE staining of the osteonecrosis of the femoral head in the Mg@Ca group at (**d1**) 4 weeks, (**d2**) 8 weeks, and (**d3**) 12 weeks. The arrows indicated the trabecular bone repair around the tunnel.

Hematoxylin-eosin and safranin O-fast green staining directly visualized bone structural organization in Mg@Ca-treated tunnels. Acidophilic bone tissue exhibited eosin affinity (red staining, Figure 5(a1–a3,b1–b3)), while basophilic components demonstrated fast green binding (blue staining, Figure 5(c1–c3,d1–d3)). Mg@Ca-treated specimens (Figure 5(b1–b3,d1–d3)) displayed progressive increases in reticular trabeculae within subcapital tunnels. Comparative analysis revealed superior trabecular density and thickness in Mg@Ca-treated tunnels relative to controls (Figure 5(a1–a3,c1–c3)), demonstrating Mg@Ca’s capacity to enhance osseous regeneration. We provide an “histology images” folder that includes more original, unformatted images in the Supplementary Materials.

## 4. Discussion

ONFH represents a prevalent orthopedic disorder characterized by ischemic bone tissue necrosis within the femoral head, followed by spontaneous repair processes and architectural alterations [3,5]. Clinical manifestations typically involve progressive pain and functional limitations, with delayed intervention potentially resulting in gait abnormalities, underscoring the critical importance of early joint-preservation strategies [47]. Hip preservation therapies aim to restore anatomical integrity and biomechanical function, thereby alleviating symptoms and enhancing patient quality of life [48]. Timely diagnosis and targeted interventions may effectively postpone the necessity for arthroplasty. Local bone regeneration constitutes a fundamental therapeutic approach for skeletal pathologies including ONFH [49], employing bioactive materials and regenerative techniques to stimulate de novo bone formation at lesion sites [14,16,21,50]. This process not only reconstructs osseous structural continuity but also restores physiological functionality, forming an integral component of comprehensive management protocols for femoral head necrosis.

We successfully synthesized an injectable assembled three-dimensional solid scaffold-structured bioceramic. Our previous investigation [46] demonstrated its superior repair efficacy for lateral condyle defects compared to autologous bone grafts, while simultaneously enhancing the proliferation and osteogenic differentiation capacity of human mesenchymal stem cells. In the osteonecrosis of the femoral head (ONFH) model, the material significantly accelerated osseous regeneration within necrotic regions, thereby establishing its potential as a promising therapeutic strategy for ONFH management.

In bone tissue engineering, advanced bioceramics serve as bioactive scaffolds releasing Mg^2+^ that act as mineral precursors and signaling activators for bone matrix regeneration. The slow and sustained release of Mg^2+^ is a key physical property of Mg@Ca. It plays a vital role in enhancing osteoblast adhesion and proliferation [51,52], improving the material’s antibacterial [53] and anti-inflammatory [52] properties, and promoting the mineralization process of osteoblasts [54]. Experimental evidence from a rat model [55] revealed that magnesium deficiency substantially impairs osteogenesis, manifesting as marked reductions in *BGLAP* and *COL1A1* mRNA expression alongside diminished osteogenic activity. Conversely, magnesium-enriched bone substitutes have been shown to significantly up-regulate the expression of osteogenic markers including *COL1A1*, *RUNX2*, and *OPN* [56,57]. Notably, magnesium incorporation in silicocarnotite systems effectively neutralizes alkaline microenvironments induced by bioceramic degradation, demonstrating remarkable cytoprotective properties [58]. In our previous experiments, we observed that a mixing ratio of culture medium to material of 10 mL:0.2 g was more conducive to cell survival rate than 10 mL:0.5 g [46]. Therefore, we selected the 10 mL:0.2 g ratio for this study.

Mg@Ca significantly enhances the expression of key osteogenic genes *COL1A1* and *BGLAP*, thereby accelerating bone regeneration. *COL1A1*, the key-encoding gene for type I collagen, is central to bone tissue formation. It gives bone structural support and regulates osteoblast proliferation and differentiation [59]. Mutations in *COL1A1* can impair bone mineralization and cause bone diseases like osteogenesis imperfecta [60], underscoring its role in skeletal homeostasis. *BGLAP* (osteocalcin) is linked to osteogenesis in multiple ways. First, it directly participates in bone formation by regulating osteoblast proliferation and differentiation, promoting bone tissue development, and enhancing bone density and quality [61]. Second, it plays a key role in skeletal energy metabolism, influencing osteogenesis-related metabolic pathways through its molecular structure, such as interacting with the Wnt signaling pathway to modulate osteoblast function [62]. Third, as a major non-collagenous bone protein, its function is regulated by various factors, including mineral-binding properties, enabling it to play a key role in coordinating metabolism between osteoblasts and osteoclasts and maintaining skeletal homeostasis [63]. It must be noted that our previous findings [46] demonstrated that Mg@Ca significantly up-regulates *ALP* expression, a critical marker gene for mineralization processes.

Let us explore in detail the osteoinduction implications of up-regulating/down-regulating genes. *BDKRB2* is closely associated with osteoarthritis. The +9/−9 bp polymorphism in its gene influences *BDKRB2* expression levels in osteoarthritis patients, subsequently affecting inflammatory responses and disease severity [64,65]. In orthopedic disorders, *PLAU* (encoding uPA) plays a critical role in macrophage-mediated bone regeneration, exhibiting high expression in bone marrow-derived macrophages [66] and being essential for the bone repair process [67]. Concurrently, *PLAU* is down-regulated during osteoclast differentiation, suggesting its potential involvement in osteoclast function. These findings indicate that *PLAU* has dual roles in bone metabolism and orthopedic diseases. *ANKRD1* serves as a key regulator in adipogenesis and osteoblastogenesis in human mesenchymal stem cells, where RNA interference-mediated suppression of *ANKRD1* promotes adipogenic differentiation while inhibiting osteoblast formation [68]. The observed down-regulation of *SCUBE3* and *S1PR1* expression induced by Mg@Ca in this study contrasts with previous reports demonstrating their pro-osteogenic effects in hMSCs [69]. This discrepancy may be attributed to the absence of the BMP2/TGF-β signaling pathway [70] and S1PR1/mitophagy axis [71] related to gene enrichment in Mg@Ca-treated samples.

Mg@Ca activates the PI3K-AKT signaling pathway, promoting osteoblast differentiation and function through multiple mechanisms. Its activation enhances osteoblast proliferation and differentiation, thereby boosting bone tissue formation and repair. For instance, the KLF2 transcription factor induces PIK3CA transcription and activates the PI3K-AKT pathway to differentiate bone marrow mesenchymal stem cells into osteoblasts [72]. Moreover, the PI3K-AKT pathway regulates osteoblast survival and function by modulating autophagy. In a study, inhibiting the PI3K-Akt/mTOR pathway induced osteoblast autophagy, highlighting its role in cellular protection under stress [73]. However, precise regulation of PI3K-AKT activity is essential, as excessive inhibition can suppress osteoblast differentiation and bone formation. For example, miR-374-5p targets PTEN to negatively regulate the PI3K-Akt pathway, thereby inhibiting osteoblast osteogenic capacity [74]. In summary, PI3K-Akt activation sustains long-term effects by modulating diverse cell populations and multiple downstream signaling pathways during osteogenesis.

The ECM-receptor interaction pathway plays a key role in Mg@Ca-mediated osteogenesis. Studies on betaine-regulated hAD-MSC differentiation show significant enrichment of this pathway alongside PI3K-Akt signaling, collectively enhancing osteoinduction through ECM-receptor-PI3K-Akt crosstalk [75]. A research on hMSCs’ osteogenic differentiation gene analysis, genes related to this pathway were found to be enriched, highlighting its importance in osteogenesis [76]. Moreover, in an osteoporosis-related multi-omics analysis, it was discovered that the expression of this pathway’s genes in BMSCs from ovariectomized rats was elevated, suggesting its potential in bone formation [77].

The metal ions dissolution dynamics of implanted bioceramics play a pivotal role in Mg@Ca-related signaling pathway. Experimental evidence demonstrates that ionic release profiles, particularly Mg^2+^ and Ca^2+^ liberation from 3D-printed bioactive porous ceramic scaffolds, critically regulate PI3K/Akt pathway activation [78]. Sodium alginate-modified calcium magnesium phosphate cement was shown to modulate PI3K/Akt biomarker expression through controlled Mg^2+^/Ca^2+^ equilibrium maintenance [79]. Furthermore, biodegradable Mg-NdZn-Zr alloy implants enhanced MC3T3-E1 osteogenic differentiation via PI3K/AKT-mediated mechanisms, as evidenced by their soluble extracts [80].

While Mg@Ca scaffolds demonstrate significant potential for bone regeneration applications, current research remains in the preliminary exploration phase. Several critical issues regarding their mechanisms of action and clinical translation prospects require further in-depth investigation. The specific mechanisms through which Mg@Ca regulates these pathways warrant further investigation. Notably, the roles of Mg^2+^ and Ca^2+^ ions released from the scaffold in modulating intracellular signaling cascades remain incompletely elucidated and represent a critical area for future research. Current limitations also include insufficient characterization of Mg@Ca’s in vivo degradation kinetics, particularly regarding local vascular perfusion at the implantation site and interfacial interactions between the material and surrounding bone tissue. Furthermore, while promising in vitro and in vivo results have been obtained, the long-term safety and efficacy of Mg@Ca in human applications require comprehensive evaluation, including assessments of its biocompatibility, degradation behavior, and potential adverse effects in clinical settings.

## 5. Conclusions

This investigation demonstrated the remarkable bone regenerative capacity of Mg@Ca self-setting particles in a femoral head osteonecrosis model, with multi-level validation spanning cellular, molecular, and in vivo assessments. Molecular analyses revealed significant up-regulation of critical osteogenic markers *COL1A1* and *BGLAP* following Mg@Ca treatment. Transcriptomic profiling identified activation of two pivotal bone regeneration pathways: PI3K-Akt signaling and ECM-receptor interactions. In vivo evaluations confirmed scaffold biodegradation concurrent with site-specific osteogenesis. The material’s injectable self-setting properties conferred exceptional washout-resistant characteristics during minimally invasive implantation. These findings collectively establish Mg@Ca self-setting particles as a clinically translatable therapeutic strategy for osteonecrosis management and bone defect reconstruction in ONFH.

## Figures and Tables

**Table 1:** Primers used for real-time PCR.

Genes	Sequences (5′-3′) Sense	Sequences (5′-3′) Antisense
*GAPDH*	ACCCACTCCTCCACCTTTGA	CATACCAGGAAATGAGCTTGACAA
*RUNX2*	ATGGCGGGTAACGATGAAA	TTGTGAAGACGGTTATGGTCAAG
*ALP*	GACCTCCTCGGAAGACACTCTG	CGCCTGGTAGTTGTTGTGAGC
*BGLAP*	GAGGGCAGCGAGGTAGTGA	TGTGGTCAGCCAACTCGTCA
*OPN*	TGGGAGGGCTTGGTTGTCA	CAGAATCAGCCTGTTTAACTGGTAT
*COL1A1*	GTGCGATGACGTGATCTGTGA	GTTTCTTGGTCGGTGGGTGA
*COL2A1*	CGCTGTCCTTCGGTGTCAG	CCTTGATGTCTCCAGGTTCTCC
*OGN*	TGAGGATAAATACCTGGAT	TGCGTAAAGATAGGCTGA
*VEGFA*	AGGGCAGAATCATCACGAAGT	AGGGTCTCGATTGGATGGCA

## Data Availability

The raw data supporting the conclusions of this article will be made available by the authors on request.

## Supplementary Materials

The following supporting information can be downloaded at: https://www.mdpi.com/article/10.3390/bioengineering12060599/s1. This article has supplementary materials including (1) histology images, (2) micro-CT images, (3) supplementary figures. Supplementary figures includes Figure S1: The XRD patterns and compressive strength of Mg@Ca. Figure S2: The Micro-CT image of the assembled Mg@Ca scaffolds. Original micro-CT and histology images of the additional rabbits (not included in the main manuscript as representative images) are added in Supplementary Materials.

## Abbreviations

The following abbreviations are used in this manuscript:

ONFH	Osteonecrosis of the Femoral Head
Mg@Ca	3D porous bioceramic scaffold combining Magnesium Silicate Hydrate with α-Hemihydrate Calcium Sulfate
α-CSH	α-calcium sulfate hemihydrate
HE	Hematoxylin-Eosin staining
h-BMSC	Human Bone Marrow Mesenchymal Stem Cell
DEGs	Differentially Expressed Genes
GSEA	Gene Set Enrichment Analysis
KEGG	Kyoto Encyclopedia of Genes and Genomes Analysis
