# Correction: Zhu et al. Injectable and Assembled Calcium Sulfate/Magnesium Silicate 3D Scaffold Promotes Bone Repair by In Situ Osteoinduction. *Bioengineering* 2025, *12*, 599

**DOI:** 10.3390/bioengineering12121375

**Published:** 2025-12-18

**Authors:** Wei Zhu, Tianhao Zhao, Han Wang, Guangli Liu, Yixin Bian, Qi Wang, Wei Xia, Siyi Cai, Xisheng Weng

**Affiliations:** 1 Department of Orthopedics, State Key Laboratory of Complex Severe and Rare Diseases, Peking Union Medical College Hospital, Chinese Academy of Medical Sciences & Peking Union Medical College, Beijing 100730, China; zhuwei9508@163.com (W.Z.); zhaoth18@student.pumc.edu.cn (T.Z.); hannah0928@hotmail.com (H.W.); 13146171903@163.com (G.L.); yxb8408@163.com (Y.B.); wangsq19@student.pumc.edu.cn (Q.W.); 2 State Key Laboratory of Complex Severe and Rare Diseases, Peking Union Medical College Hospital, Chinese Academy of Medical Sciences & Peking Union Medical College, Beijing 100730, China; 3 Centro Médico de Macau do Peking Union Medical College Hospital, Macau 999078, China; 4 Applied Materials Science, Department of Materials Science and Engineering, Uppsala University, 752 37 Uppsala, Sweden; wei.xia@angstrom.uu.se

## Affiliation Revision

In the original publication [1], there was an error regarding the order of the affiliations. Affiliation 1 and affiliation 2 were swapped.

Affiliation 1 has now been updated to “Department of Orthopedics, State Key Laboratory of Complex Severe and Rare Diseases, Peking Union Medical College Hospital, Chinese Academy of Medical Sciences & Peking Union Medical College, Beijing 100730, China”. Affiliation 2 has now been updated to “State Key Laboratory of Complex Severe and Rare Diseases, Peking Union Medical College Hospital, Chinese Academy of Medical Sciences & Peking Union Medical College, Beijing 100730, China”.

Moreover, in the original publication [1], the affiliation for the author Dr. Siyi Cai was mistakenly indicated as affiliation 4, instead of the original affiliation 2 (i.e., affiliation 1 in the corrected paper). The affiliation of Dr. Siyi Cai has now been updated as 1: “Department of Orthopedics, State Key Laboratory of Complex Severe and Rare Diseases, Peking Union Medical College Hospital, Chinese Academy of Medical Sciences & Peking Union Medical College, Beijing 100730, China”.

The authors state that the scientific conclusions are unaffected. This correction was approved by the Academic Editor. The original publication has also been updated.
