# Supplementary figures and images for: Injectable and Assembled Calcium Sulfate/Magnesium Silicate 3D Scaffold Promotes Bone Repair by In Situ Osteoinduction
Source: Bioengineering (Basel). 2025 May 31;12(6):599. doi: 10.3390/bioengineering12060599 (PMC12190161; doi:10.3390/bioengineering12060599)

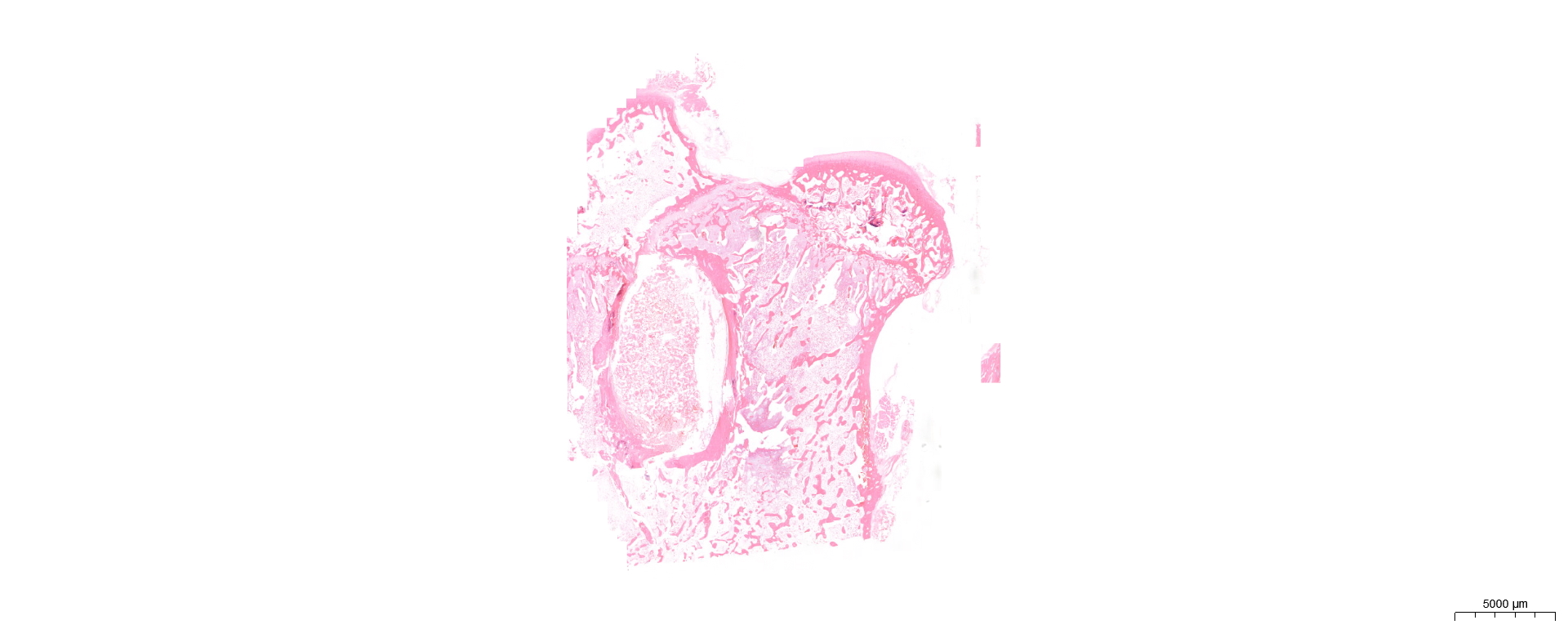

Supplement: Supplementary file 1 [file bioengineering-12-00599-s001.zip › supplementary materials/histology images/HE-Control/12 weeks 1.jpg]

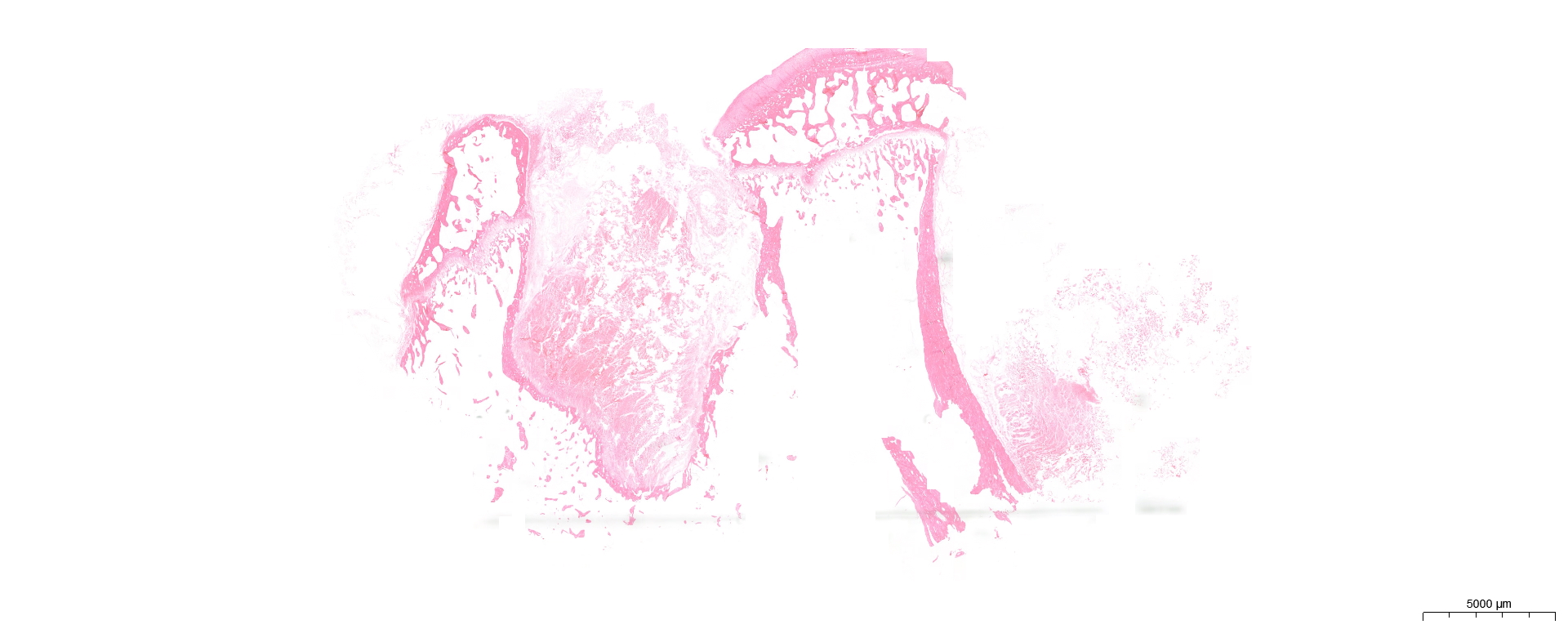

Supplement: Supplementary file 1 [file bioengineering-12-00599-s001.zip › supplementary materials/histology images/HE-Control/4 weeks 1.jpg]

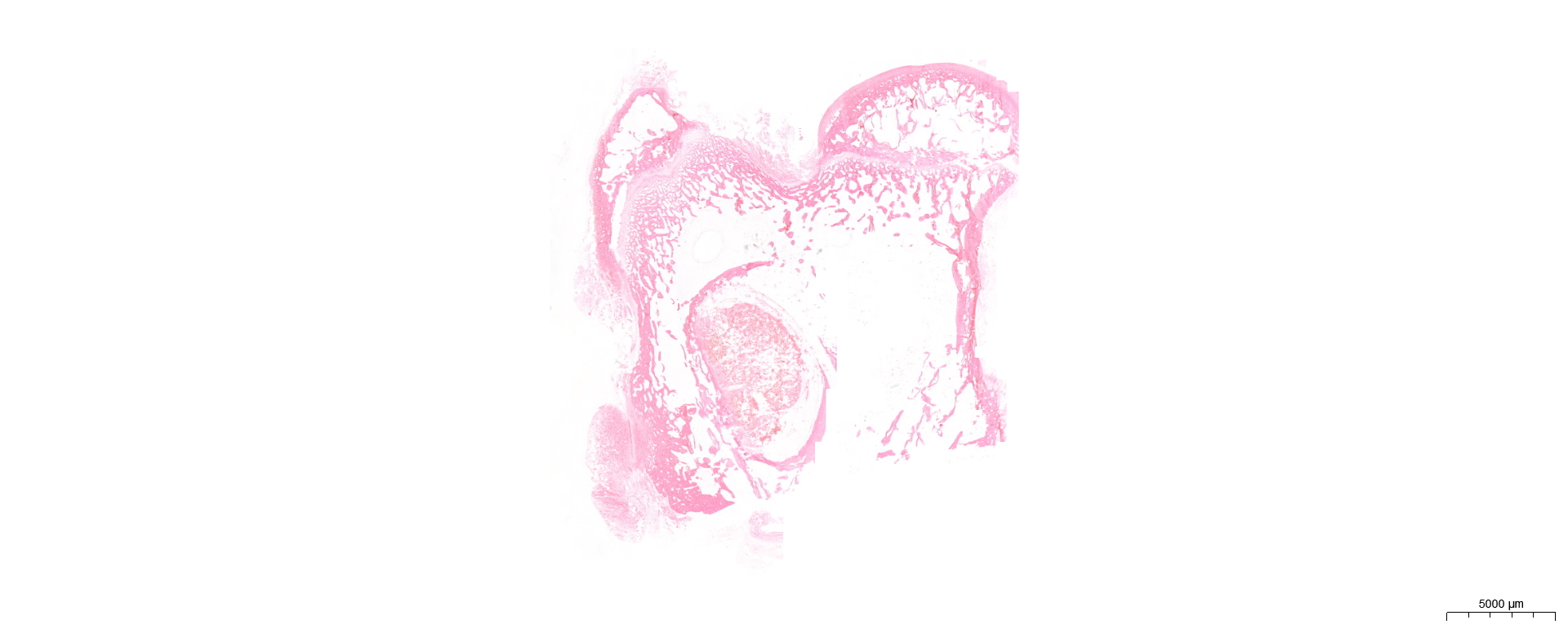

Supplement: Supplementary file 1 [file bioengineering-12-00599-s001.zip › supplementary materials/histology images/HE-Control/8 weeks 1.jpg]

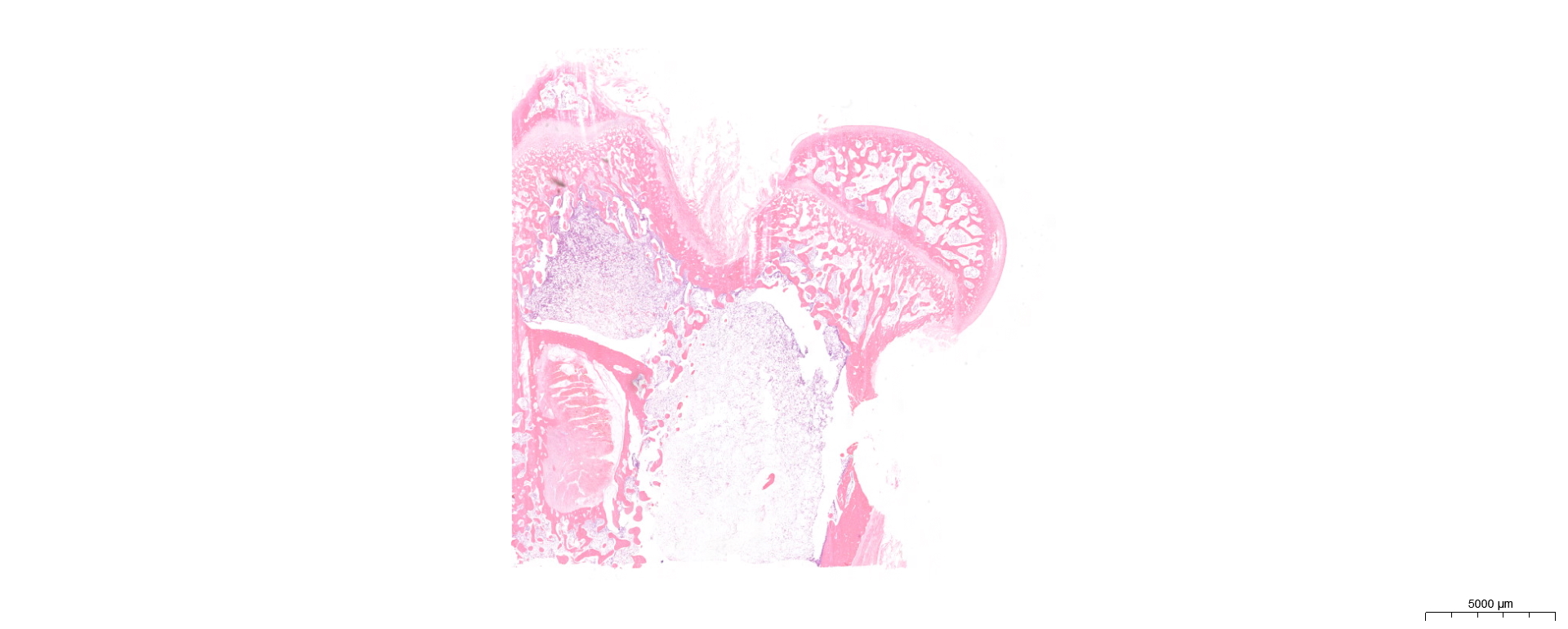

Supplement: Supplementary file 1 [file bioengineering-12-00599-s001.zip › supplementary materials/histology images/HE-Mg@Ca/12 weeks 1.jpg]

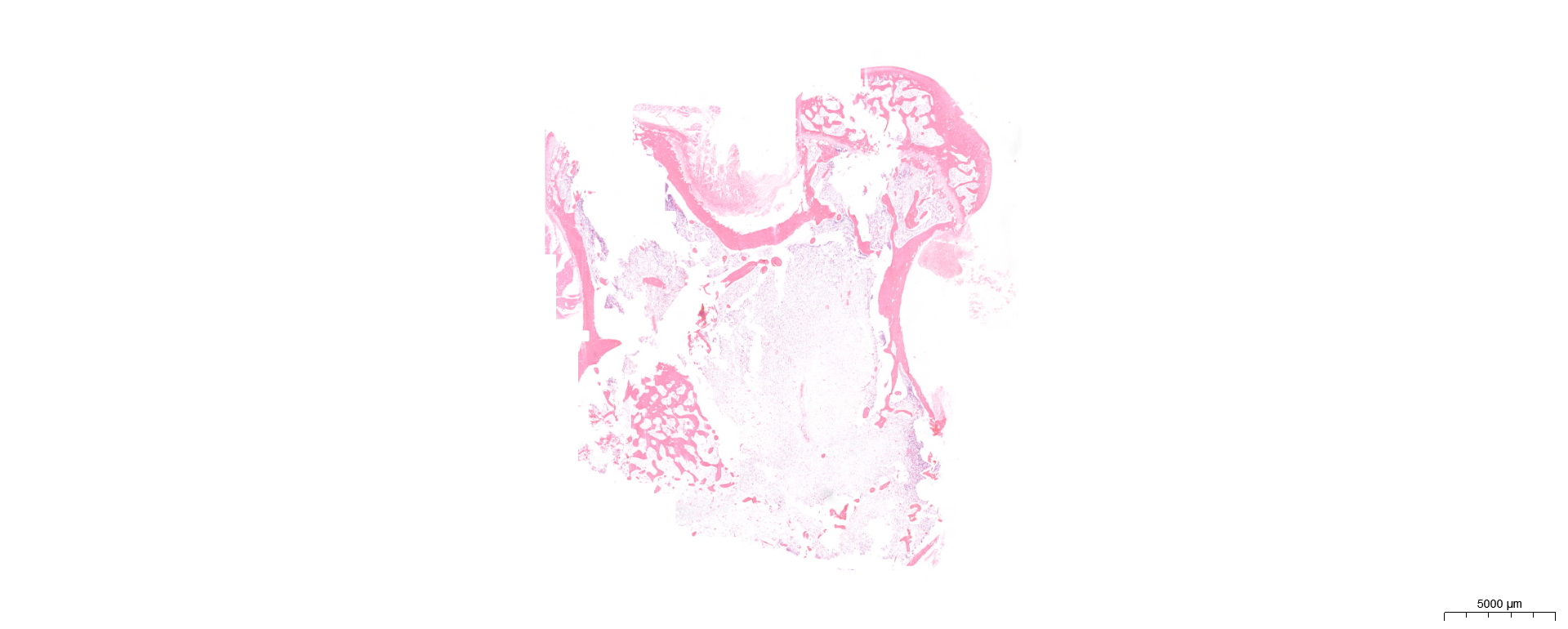

Supplement: Supplementary file 1 [file bioengineering-12-00599-s001.zip › supplementary materials/histology images/HE-Mg@Ca/4 weeks 1.jpg]

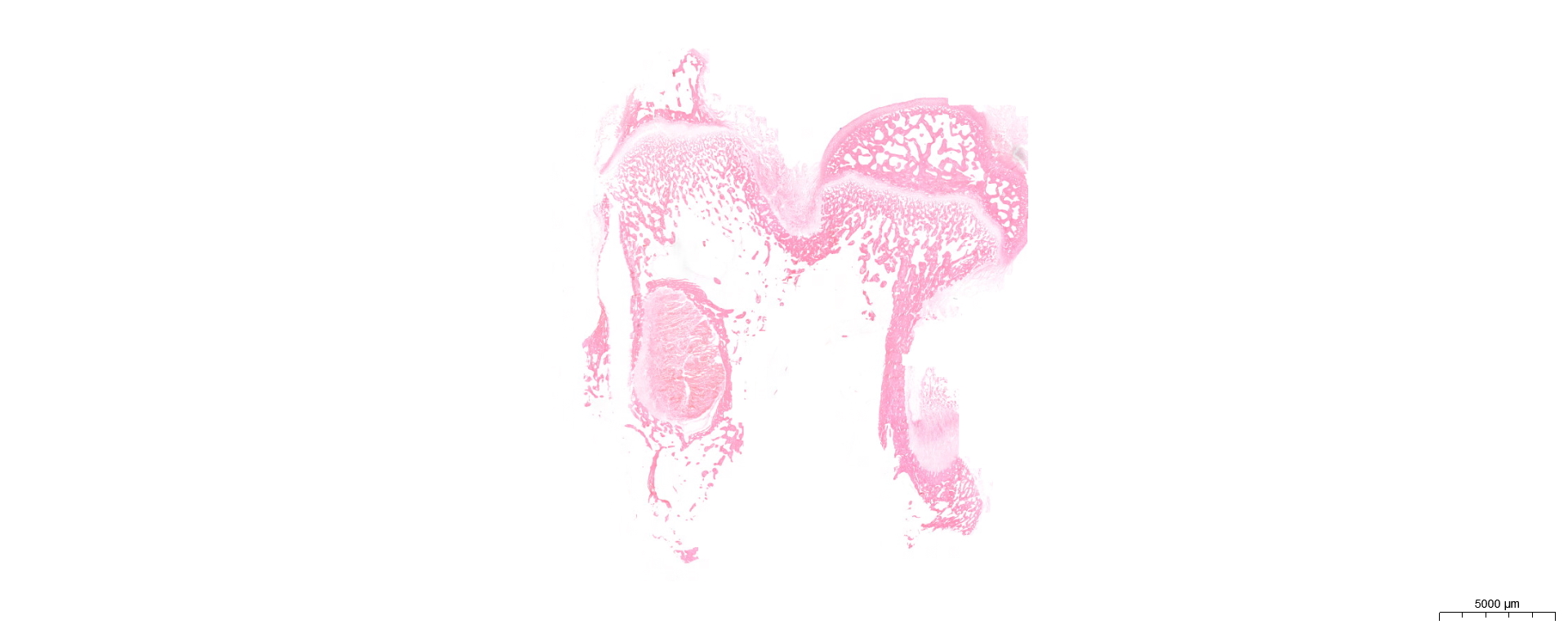

Supplement: Supplementary file 1 [file bioengineering-12-00599-s001.zip › supplementary materials/histology images/HE-Mg@Ca/8 weeks 1.jpg]

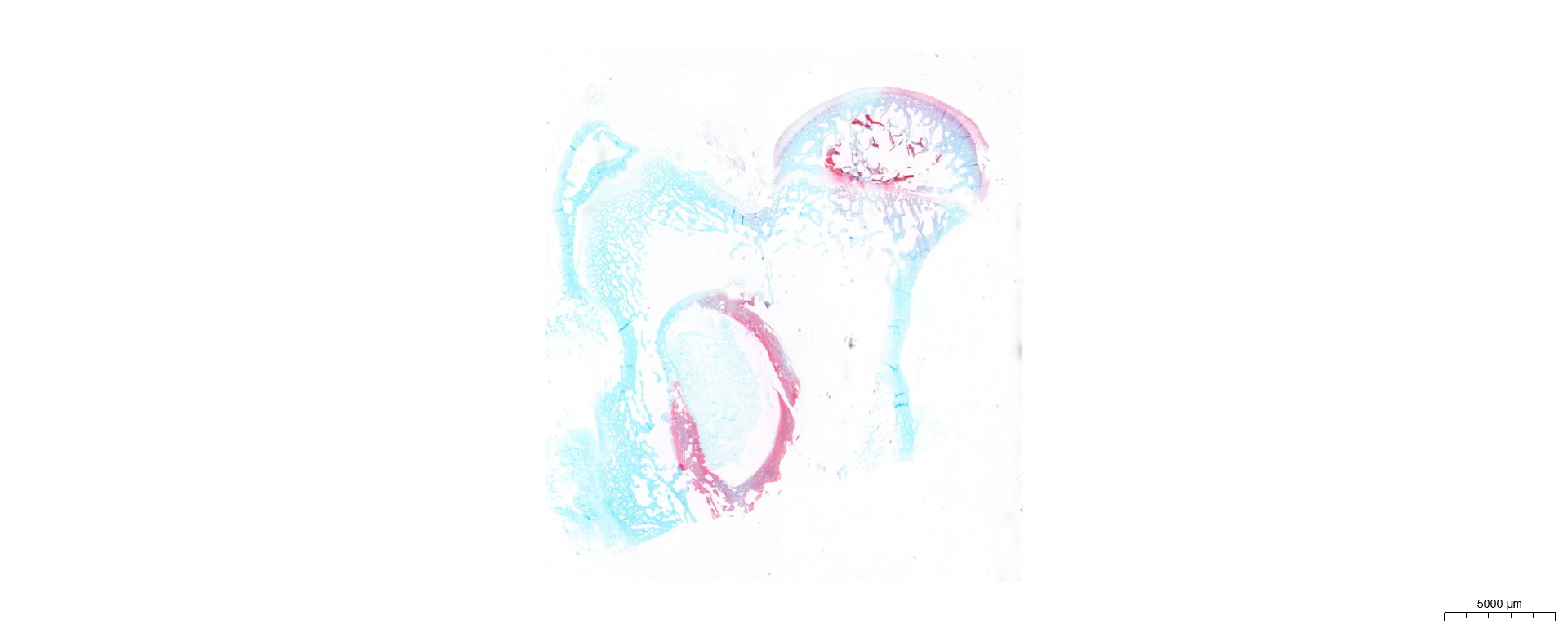

Supplement: Supplementary file 1 [file bioengineering-12-00599-s001.zip › supplementary materials/histology images/safranin O-fast green - Control/12 weeks 1.jpg]

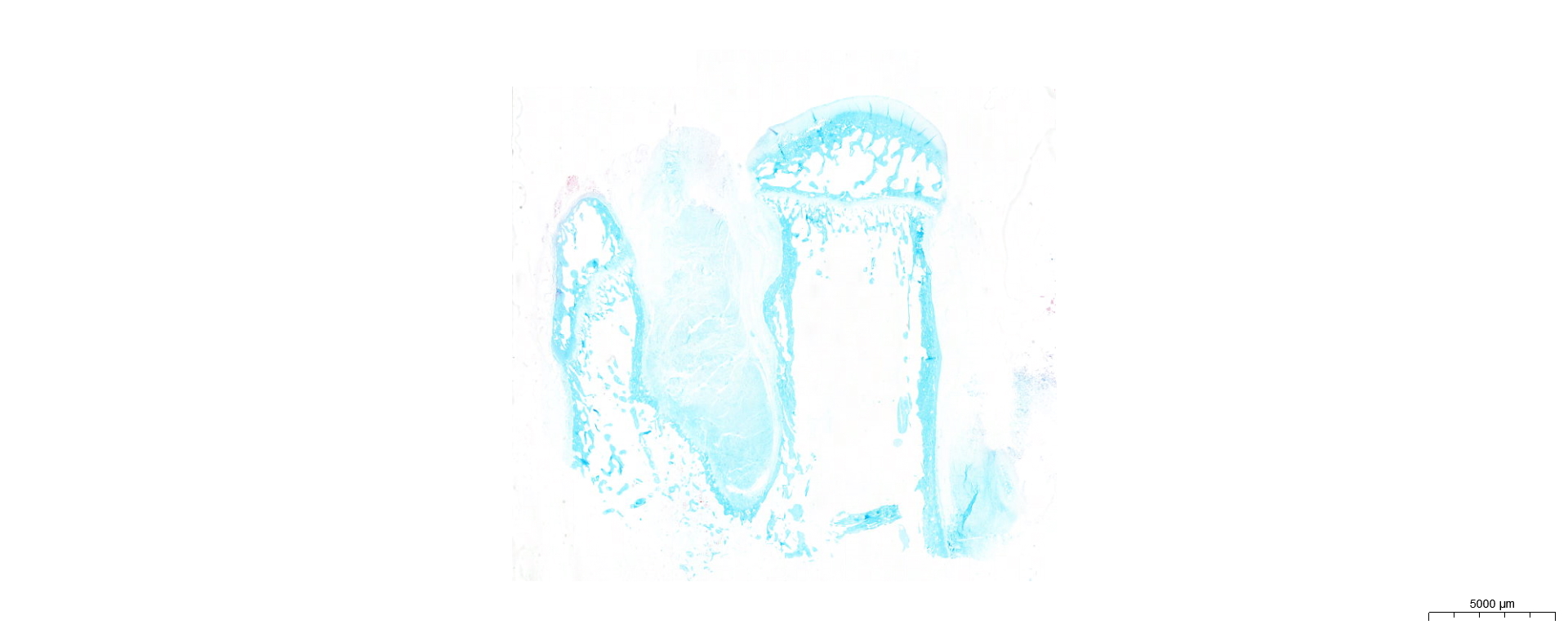

Supplement: Supplementary file 1 [file bioengineering-12-00599-s001.zip › supplementary materials/histology images/safranin O-fast green - Control/4 week 1.jpg]

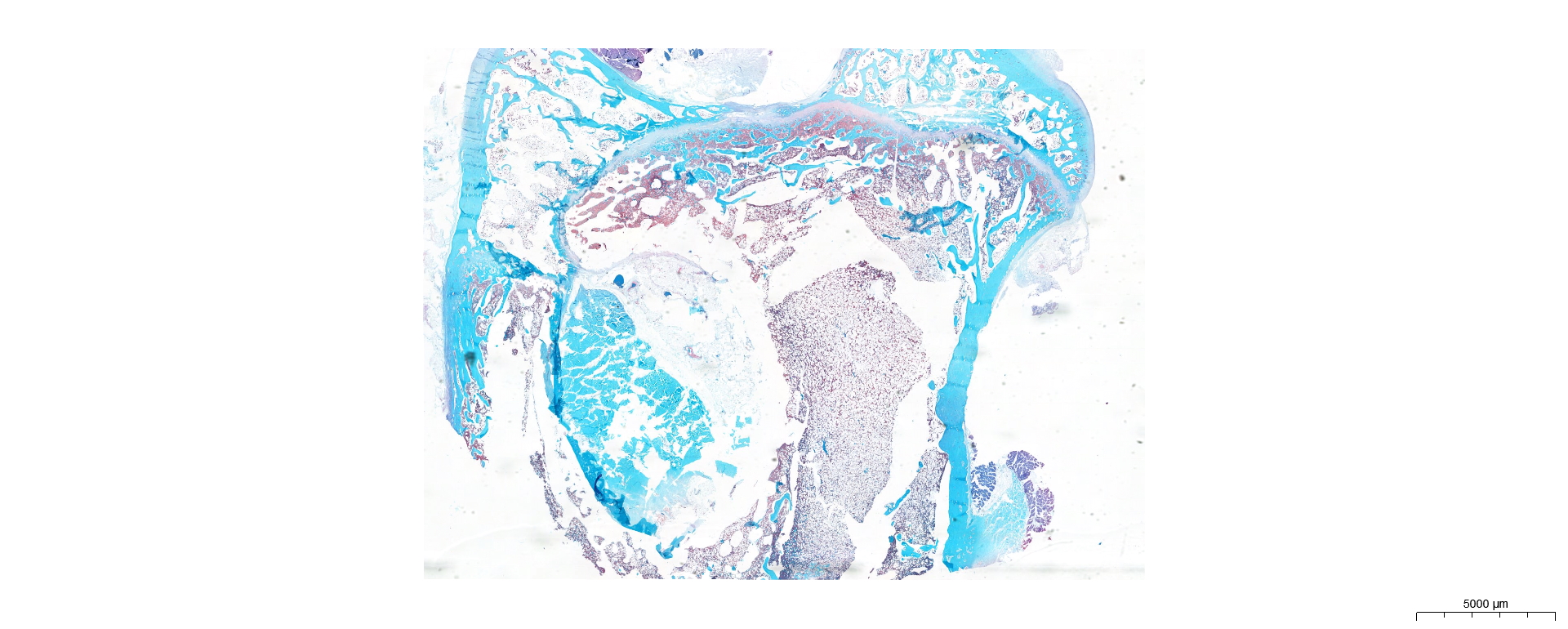

Supplement: Supplementary file 1 [file bioengineering-12-00599-s001.zip › supplementary materials/histology images/safranin O-fast green - Control/8 weeks 1.jpg]

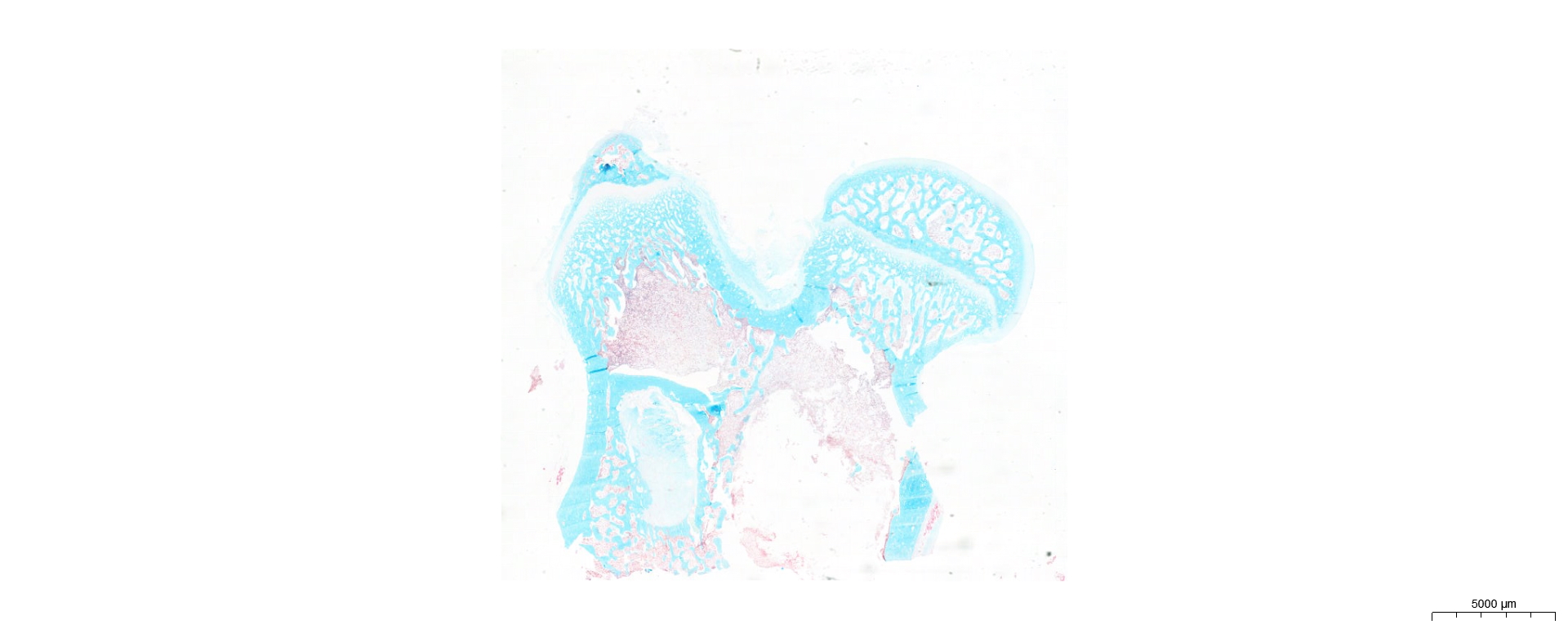

Supplement: Supplementary file 1 [file bioengineering-12-00599-s001.zip › supplementary materials/histology images/safranin O-fast green - Mg@Ca/12 weeks 1.jpg]

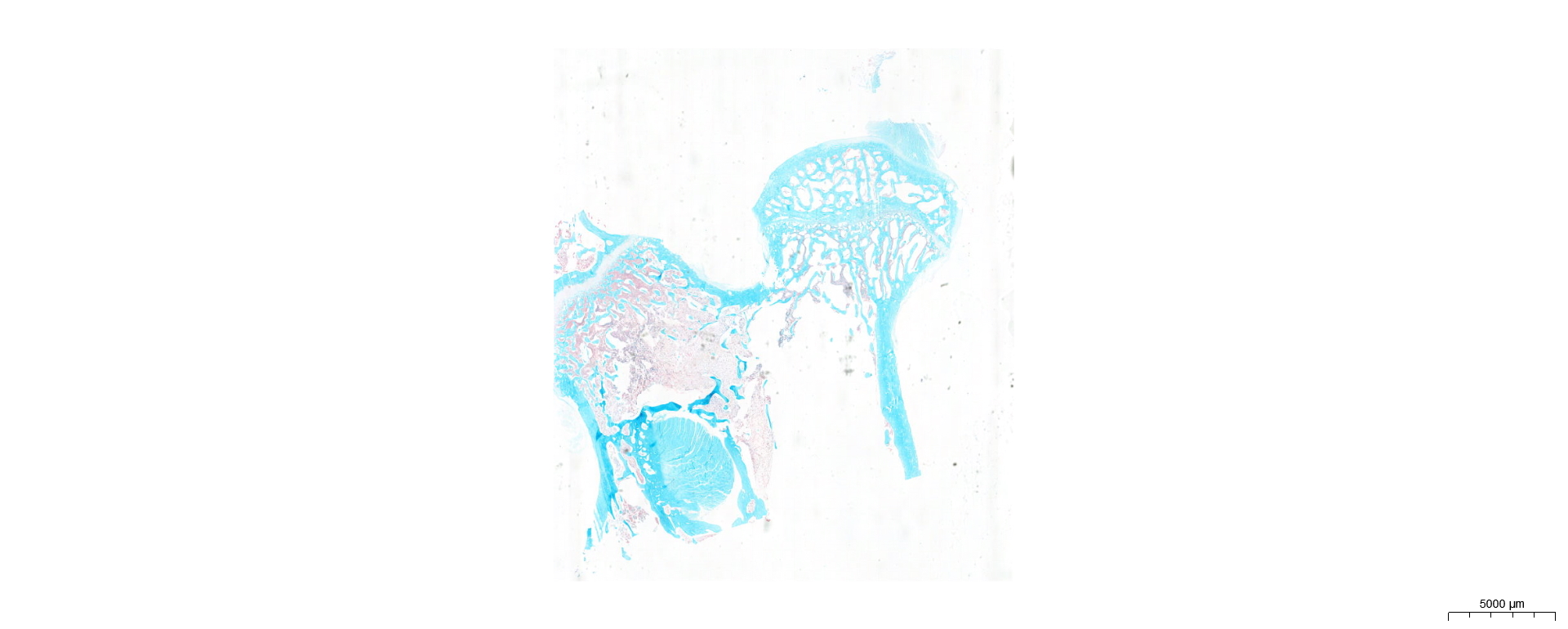

Supplement: Supplementary file 1 [file bioengineering-12-00599-s001.zip › supplementary materials/histology images/safranin O-fast green - Mg@Ca/4 weeks 1.jpg]

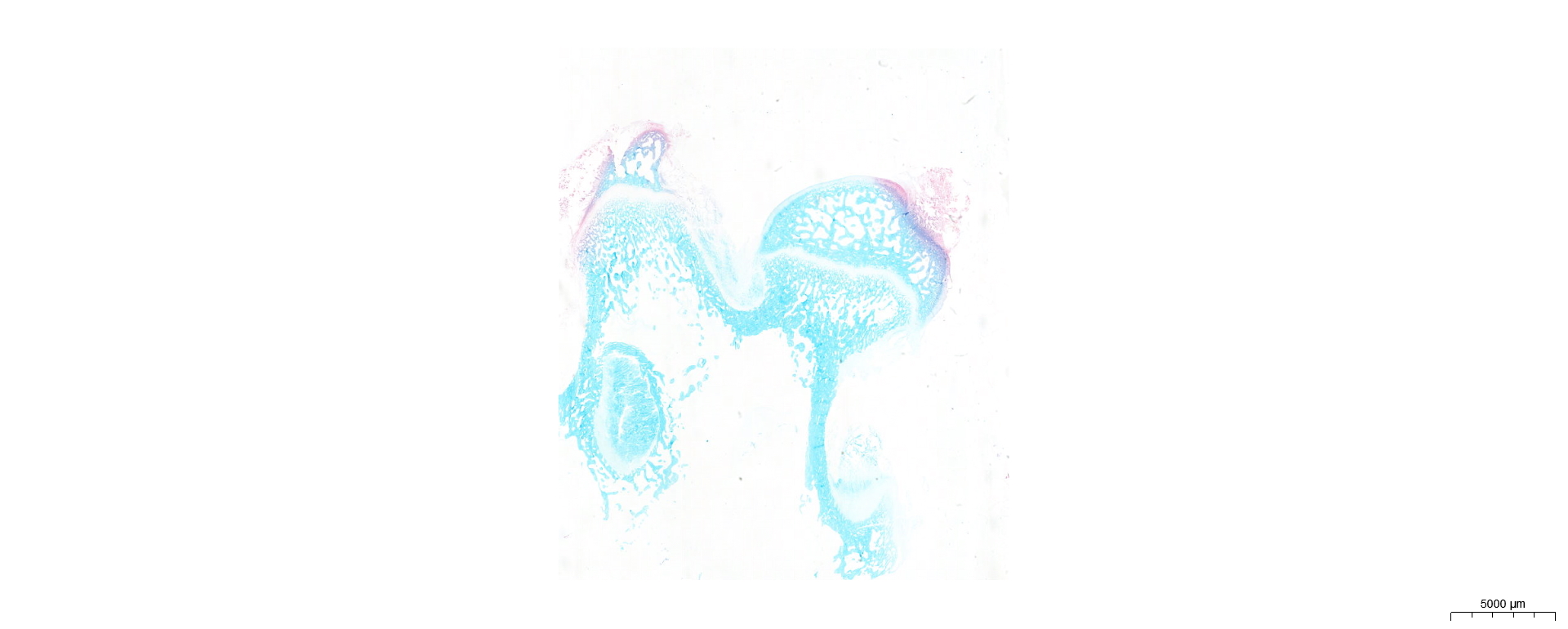

Supplement: Supplementary file 1 [file bioengineering-12-00599-s001.zip › supplementary materials/histology images/safranin O-fast green - Mg@Ca/8 weeks 1.jpg]

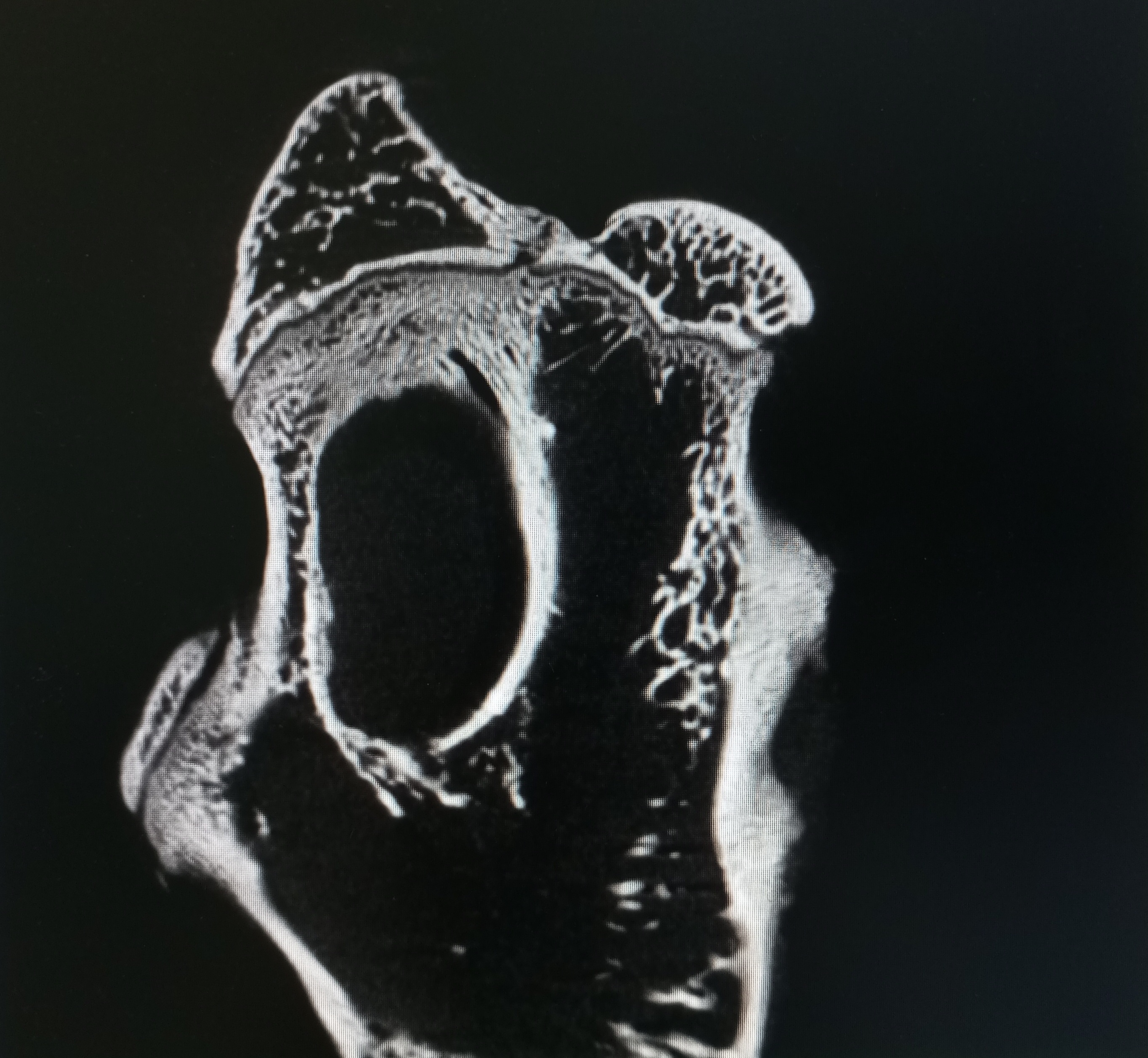

Supplement: Supplementary file 1 [file bioengineering-12-00599-s001.zip › supplementary materials/micro-CT images/control/12 weeks 1-1.jpg]

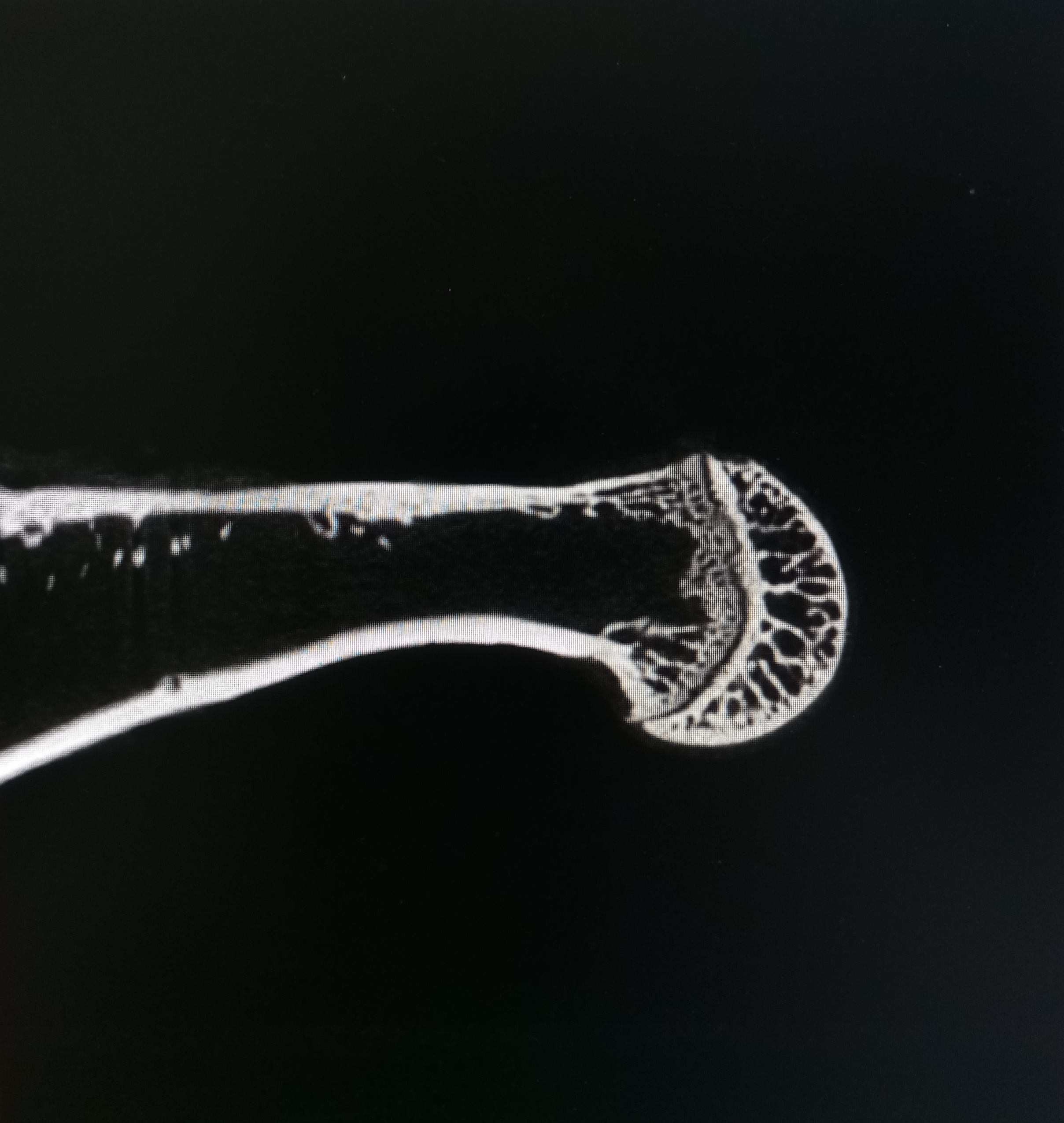

Supplement: Supplementary file 1 [file bioengineering-12-00599-s001.zip › supplementary materials/micro-CT images/control/12 weeks 1-2.jpg]

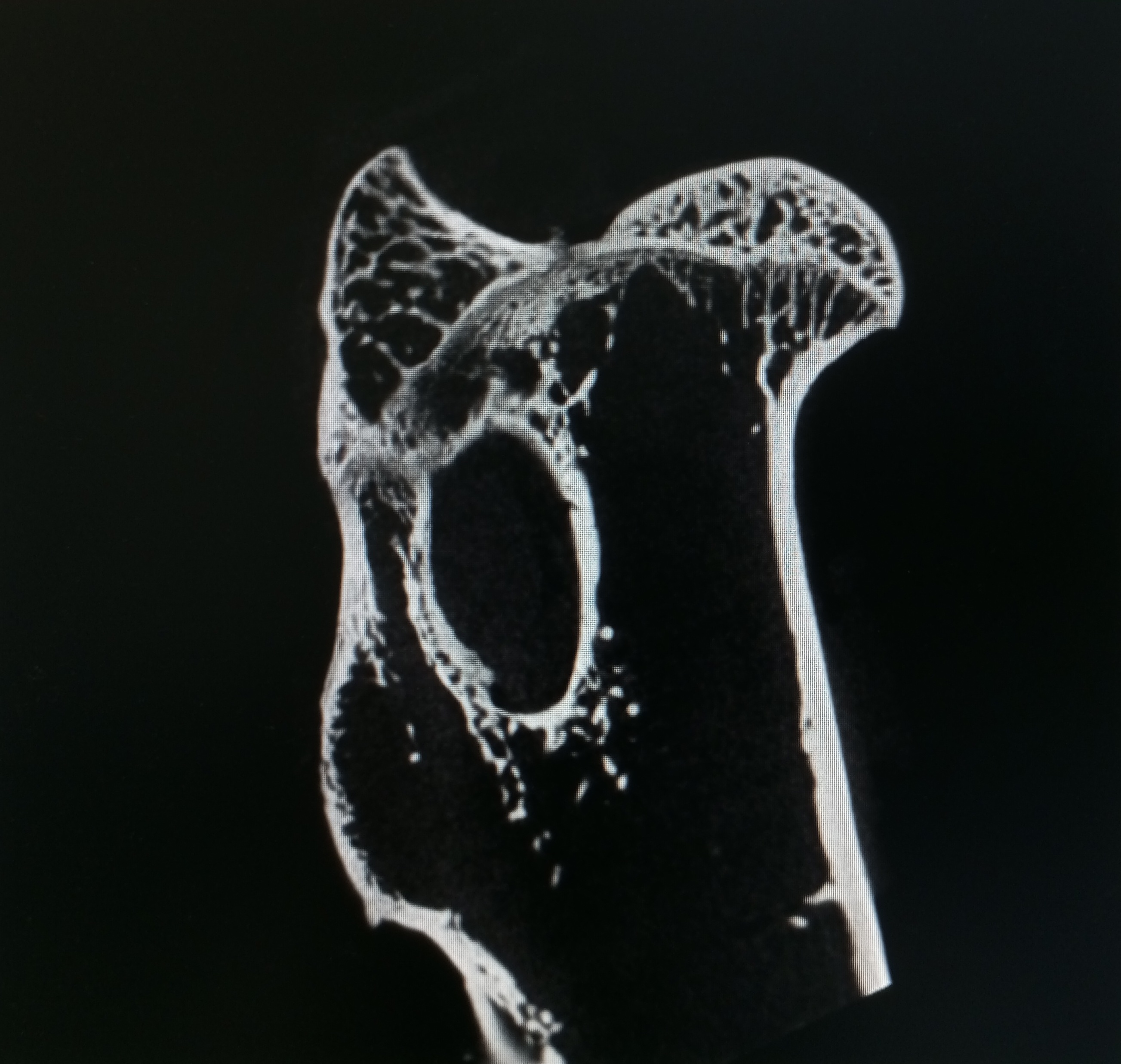

Supplement: Supplementary file 1 [file bioengineering-12-00599-s001.zip › supplementary materials/micro-CT images/control/12 weeks 2-1.jpg]

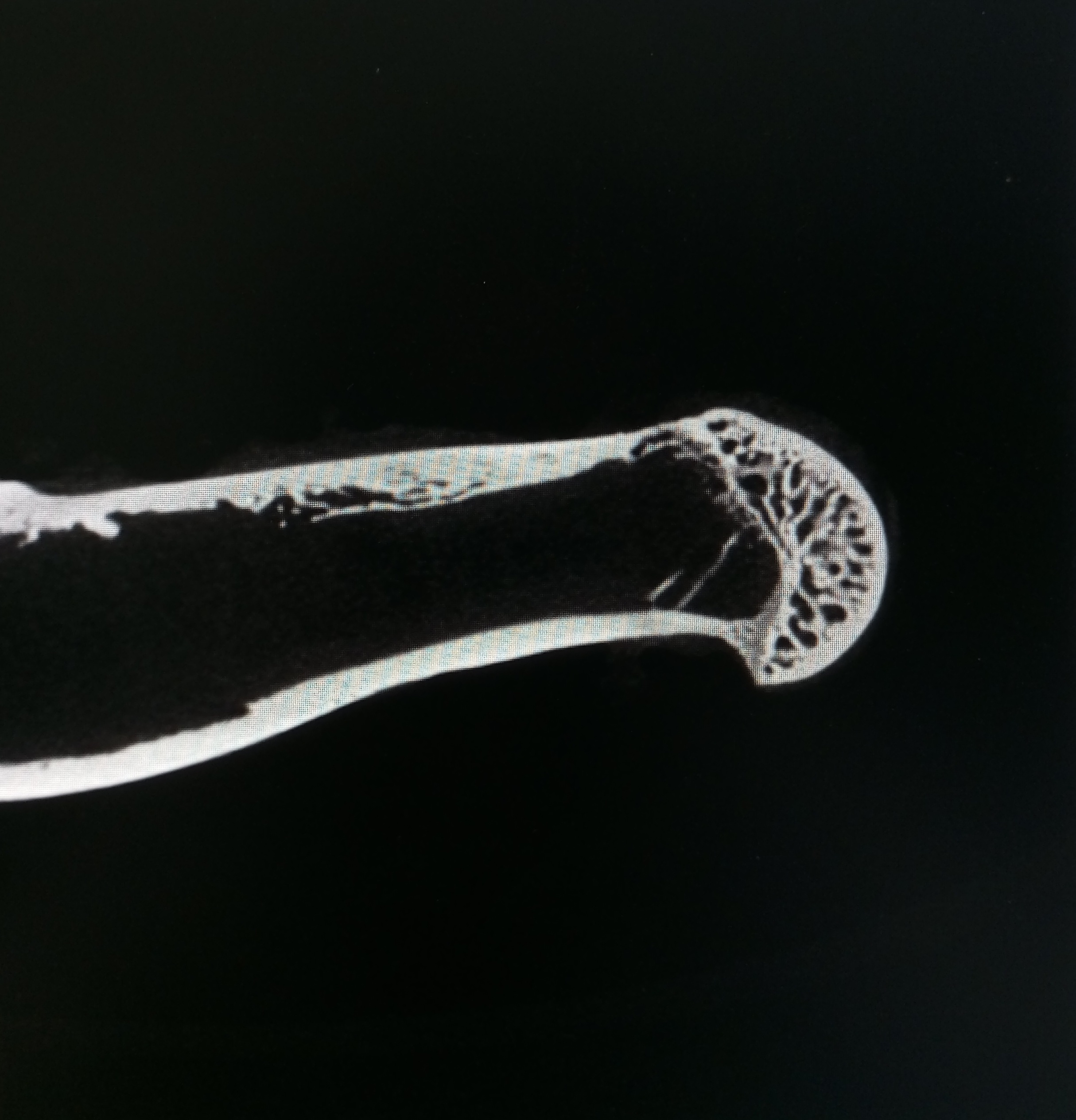

Supplement: Supplementary file 1 [file bioengineering-12-00599-s001.zip › supplementary materials/micro-CT images/control/12 weeks 2-2.jpg]

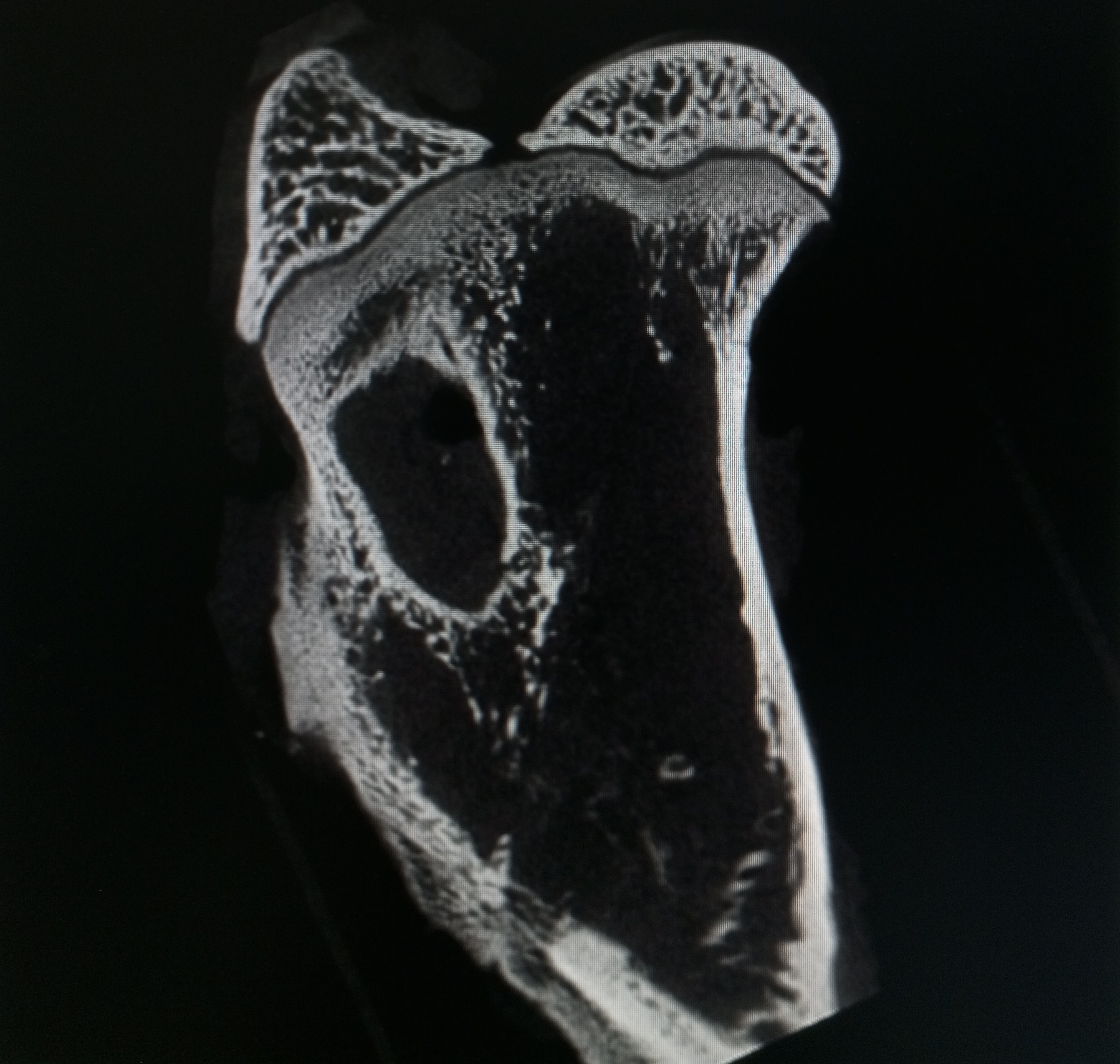

Supplement: Supplementary file 1 [file bioengineering-12-00599-s001.zip › supplementary materials/micro-CT images/control/4 weeks 1-1.jpg]

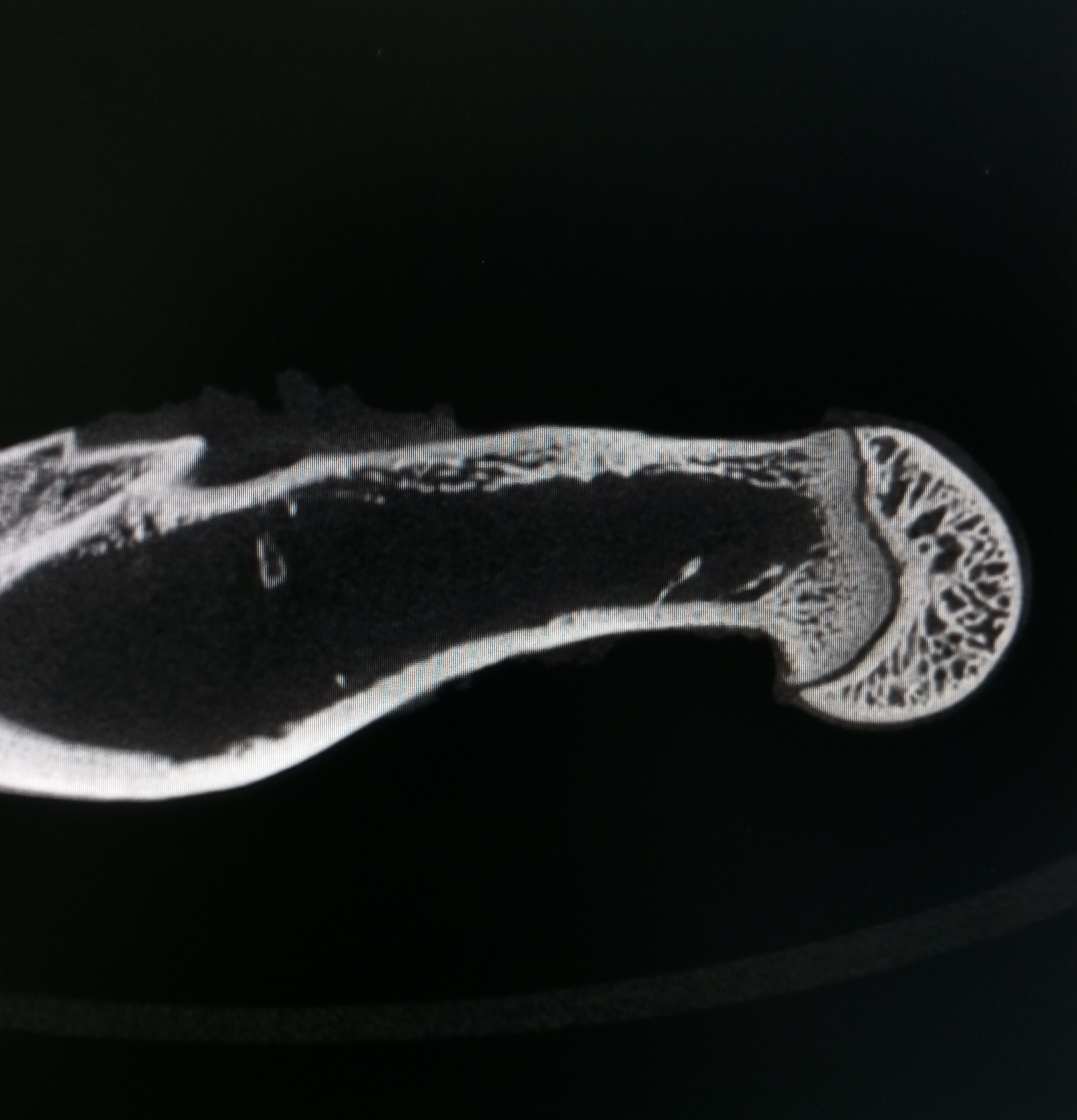

Supplement: Supplementary file 1 [file bioengineering-12-00599-s001.zip › supplementary materials/micro-CT images/control/4 weeks 1-2.jpg]

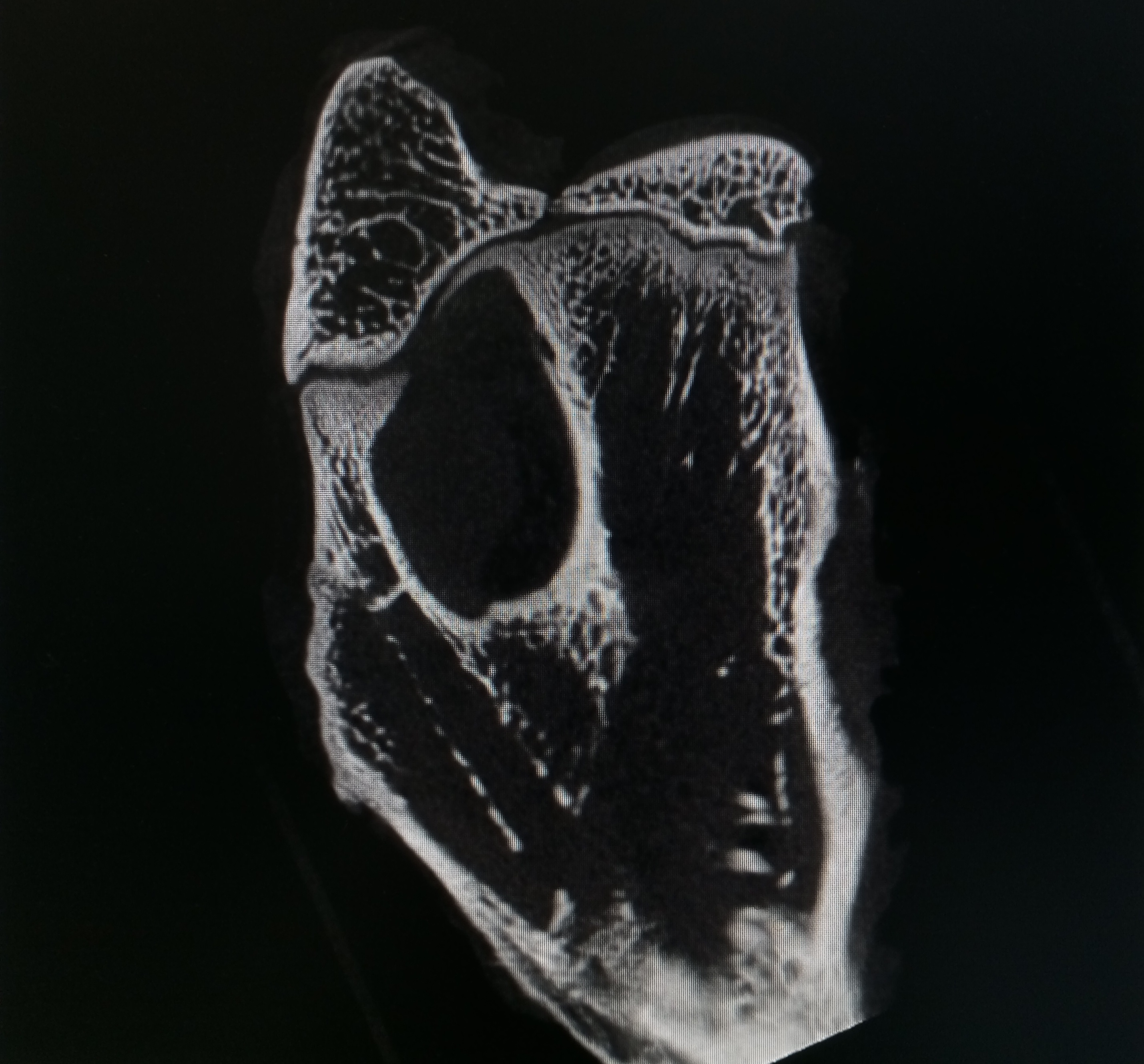

Supplement: Supplementary file 1 [file bioengineering-12-00599-s001.zip › supplementary materials/micro-CT images/control/4 weeks 2-1.jpg]

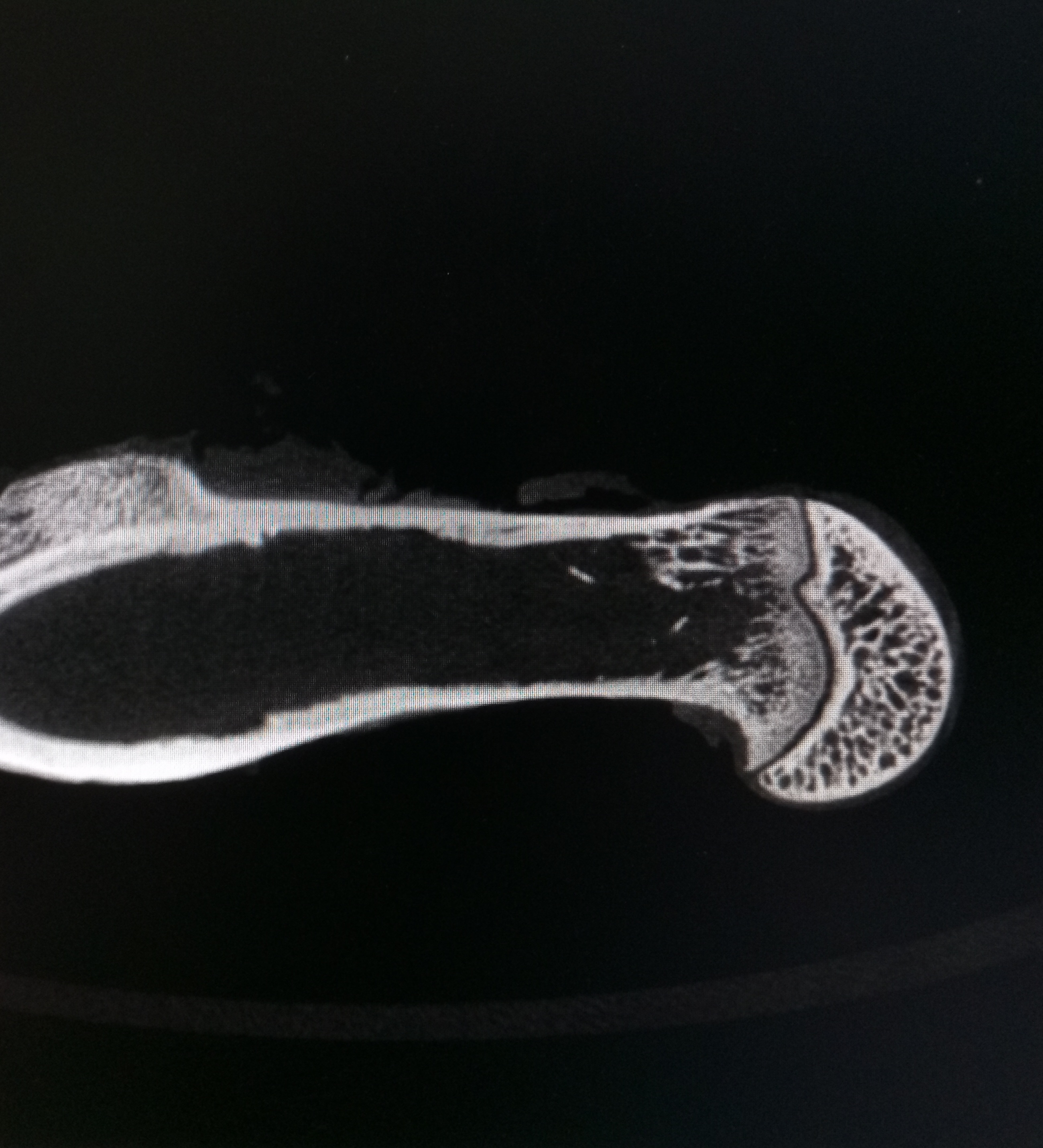

Supplement: Supplementary file 1 [file bioengineering-12-00599-s001.zip › supplementary materials/micro-CT images/control/4 weeks 2-2.jpg]

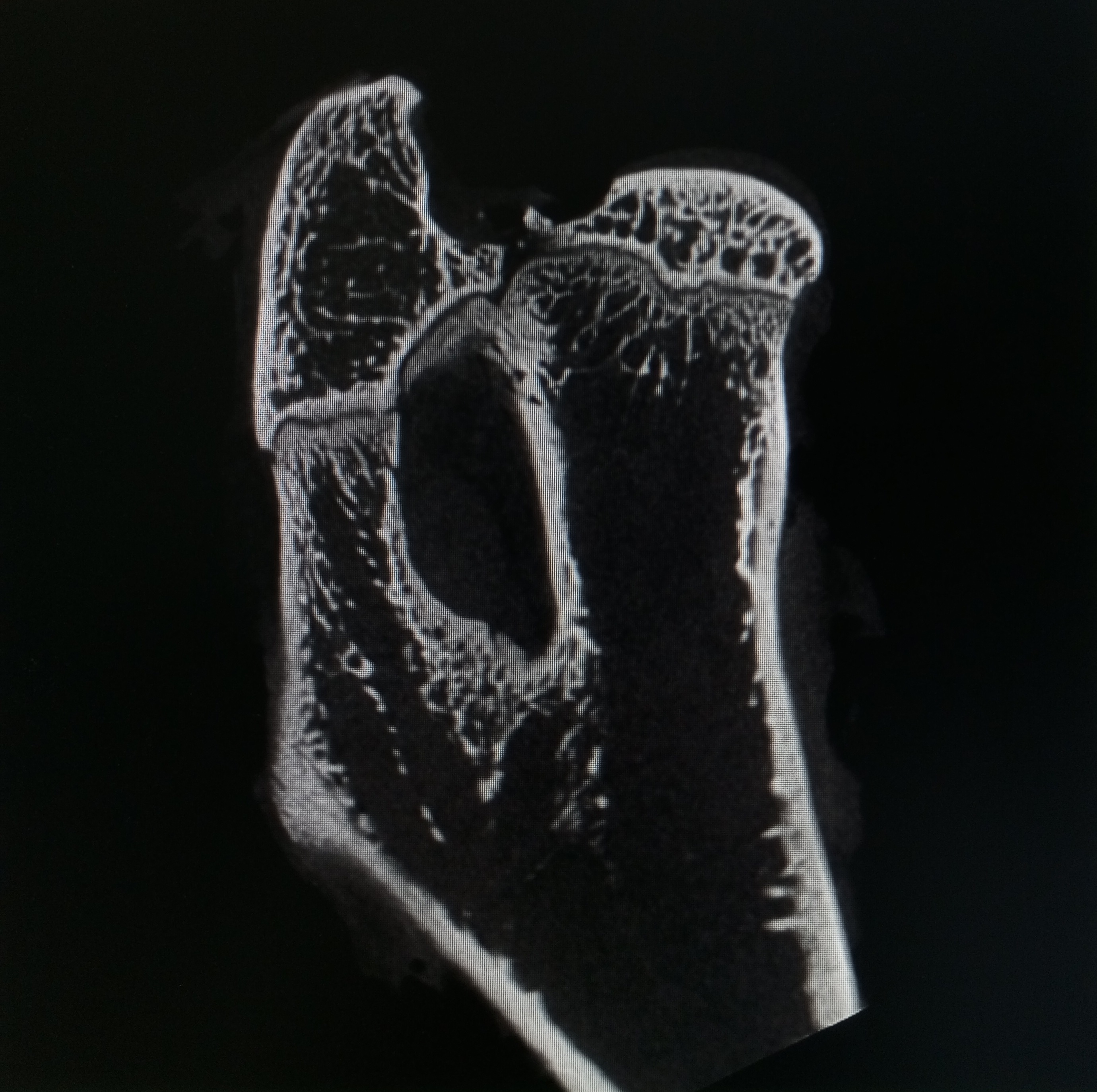

Supplement: Supplementary file 1 [file bioengineering-12-00599-s001.zip › supplementary materials/micro-CT images/control/8 weeks 1-1.jpg]

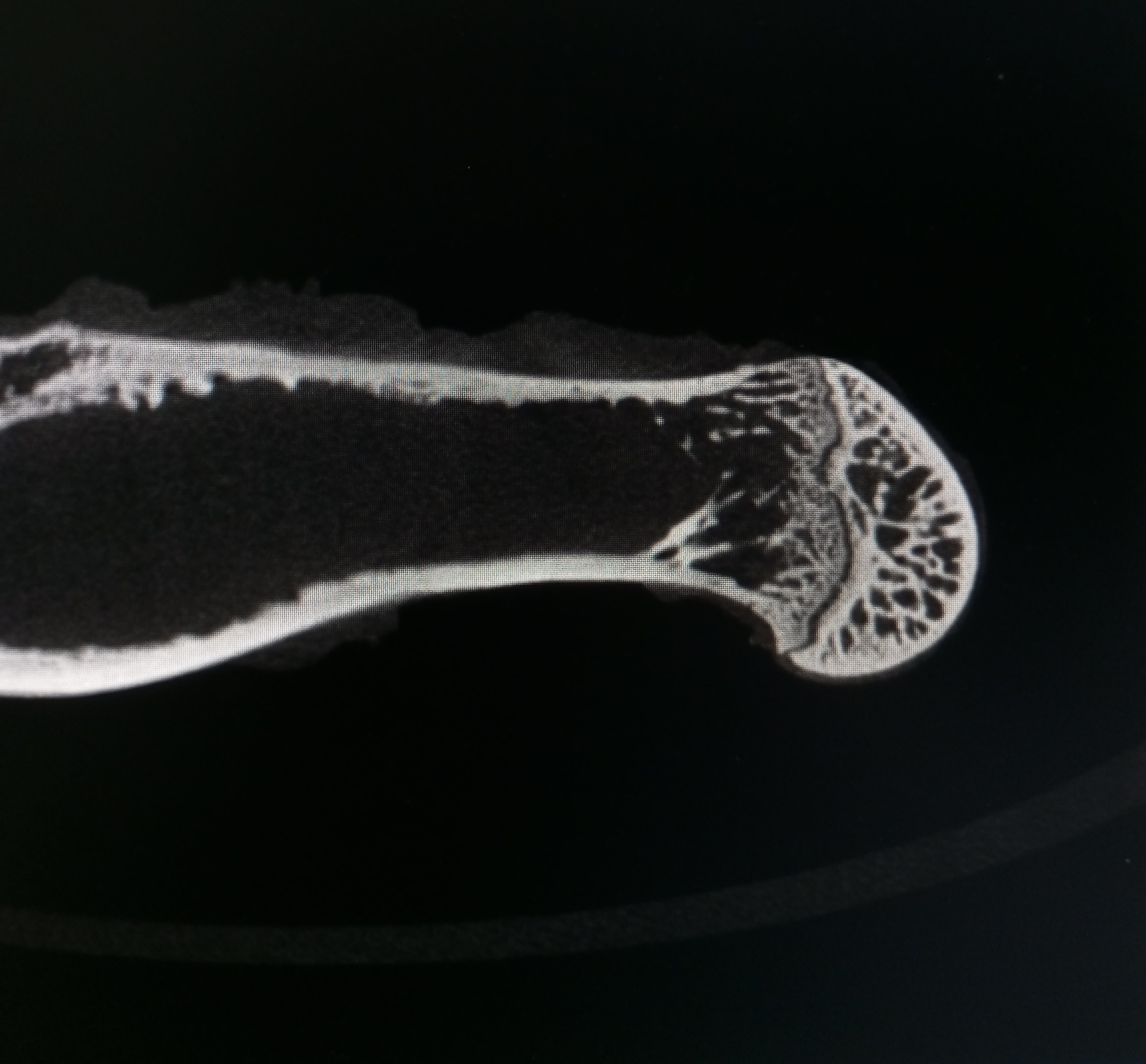

Supplement: Supplementary file 1 [file bioengineering-12-00599-s001.zip › supplementary materials/micro-CT images/control/8 weeks 1-2.jpg]

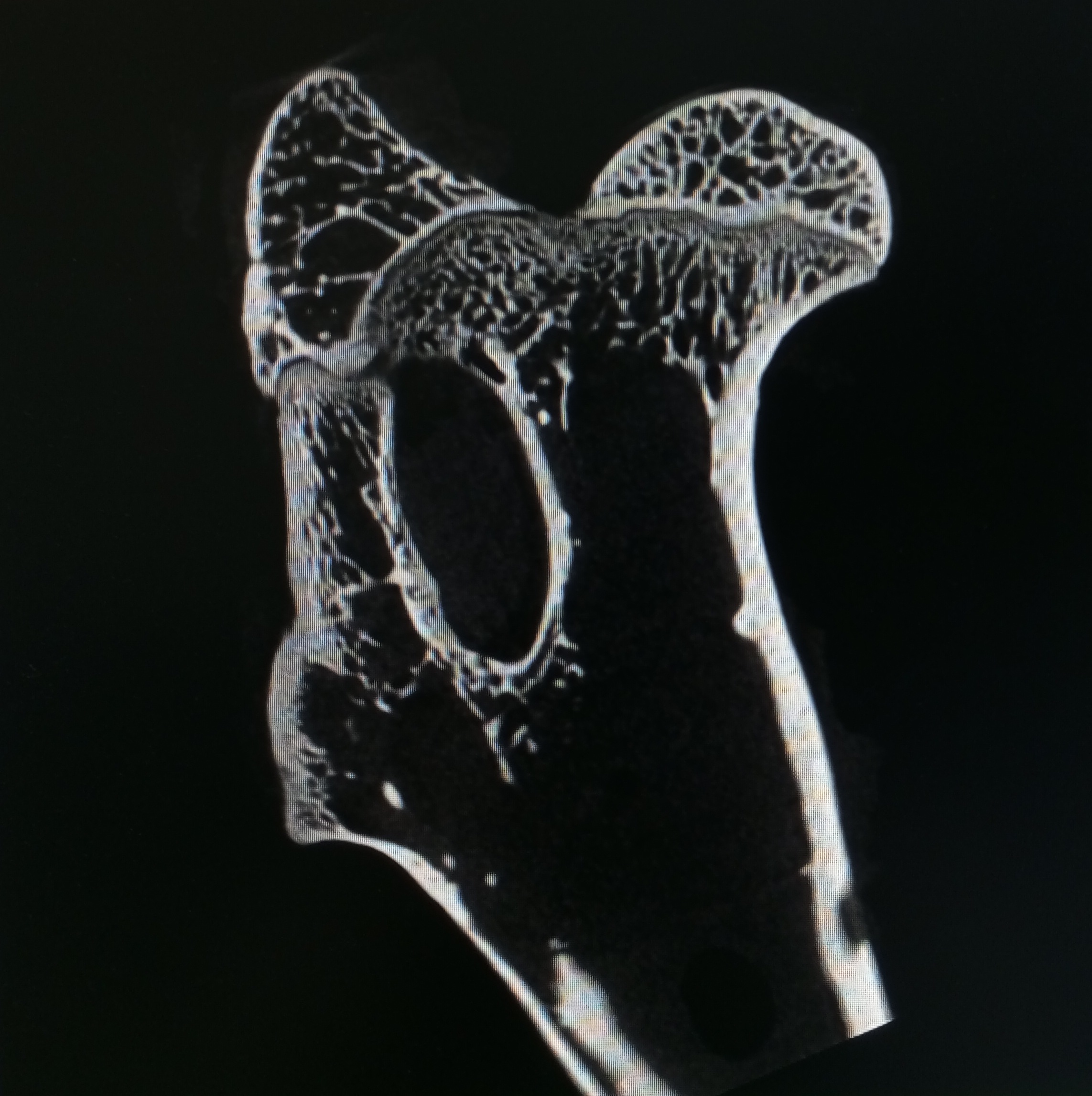

Supplement: Supplementary file 1 [file bioengineering-12-00599-s001.zip › supplementary materials/micro-CT images/control/8 weeks 2-1.jpg]

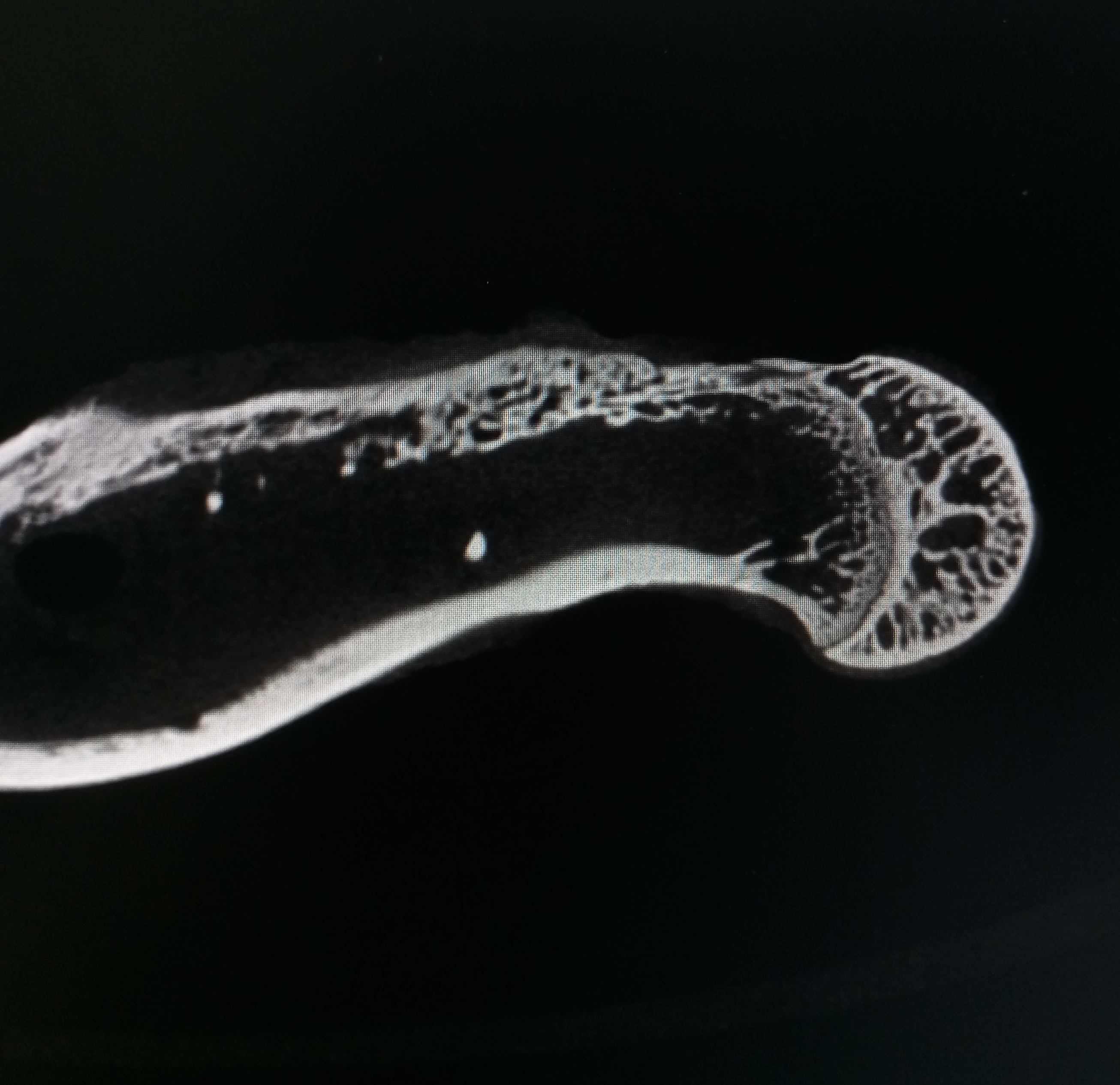

Supplement: Supplementary file 1 [file bioengineering-12-00599-s001.zip › supplementary materials/micro-CT images/control/8 weeks 2-2.jpg]

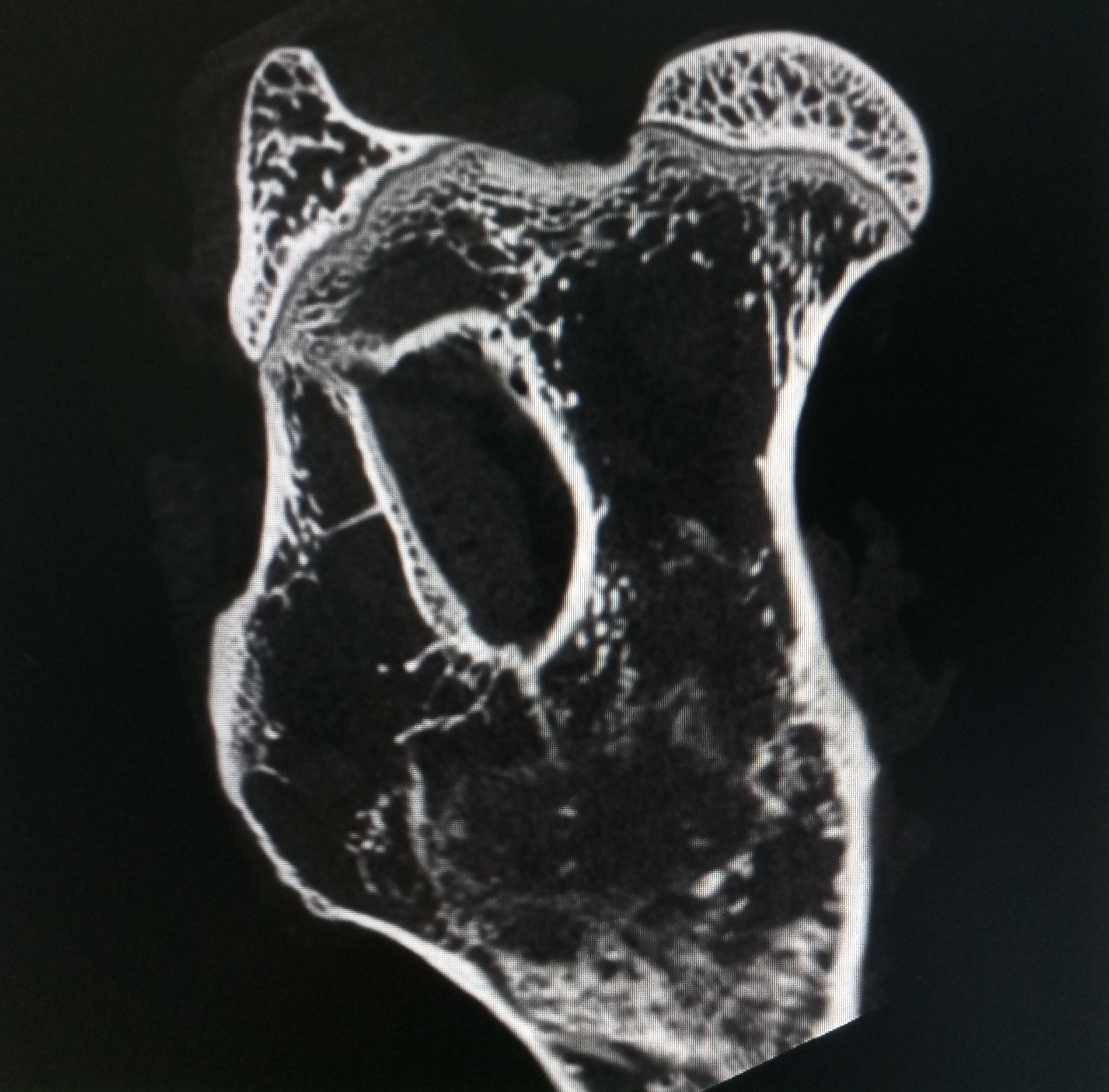

Supplement: Supplementary file 1 [file bioengineering-12-00599-s001.zip › supplementary materials/micro-CT images/Mg@Ca/12 weeks 1.jpg]

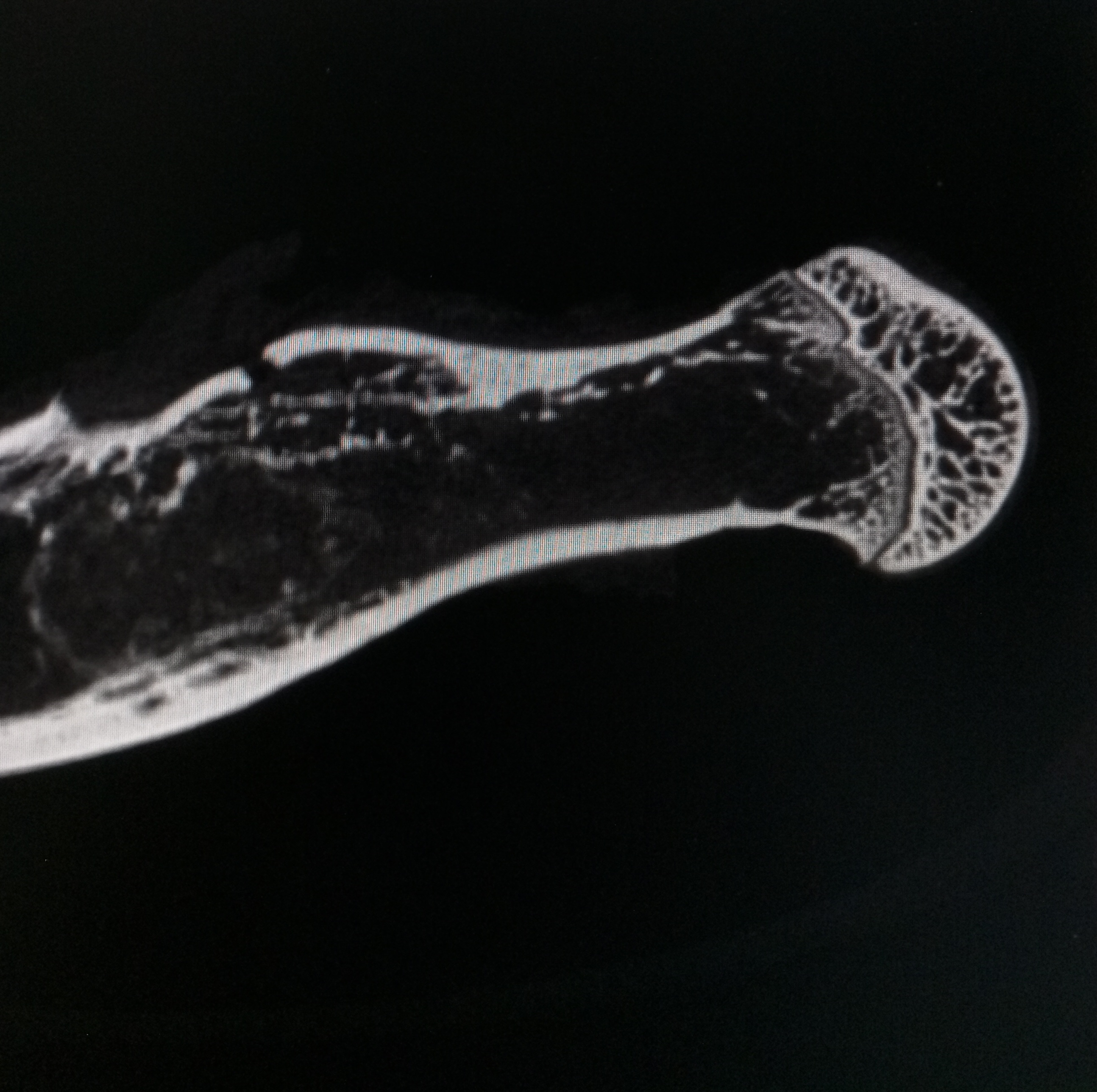

Supplement: Supplementary file 1 [file bioengineering-12-00599-s001.zip › supplementary materials/micro-CT images/Mg@Ca/12 weeks 2.jpg]

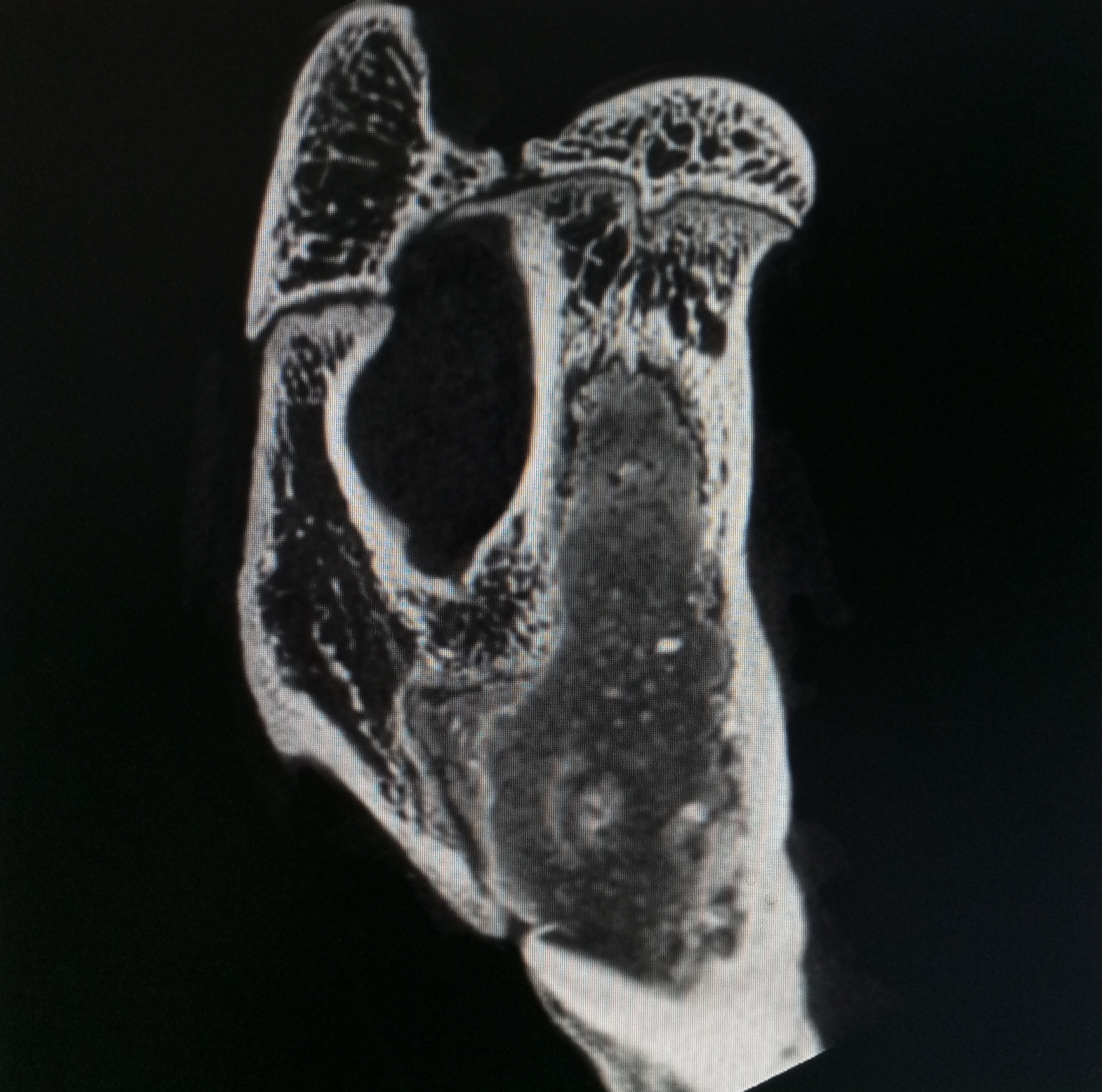

Supplement: Supplementary file 1 [file bioengineering-12-00599-s001.zip › supplementary materials/micro-CT images/Mg@Ca/4 weeks 1.jpg]

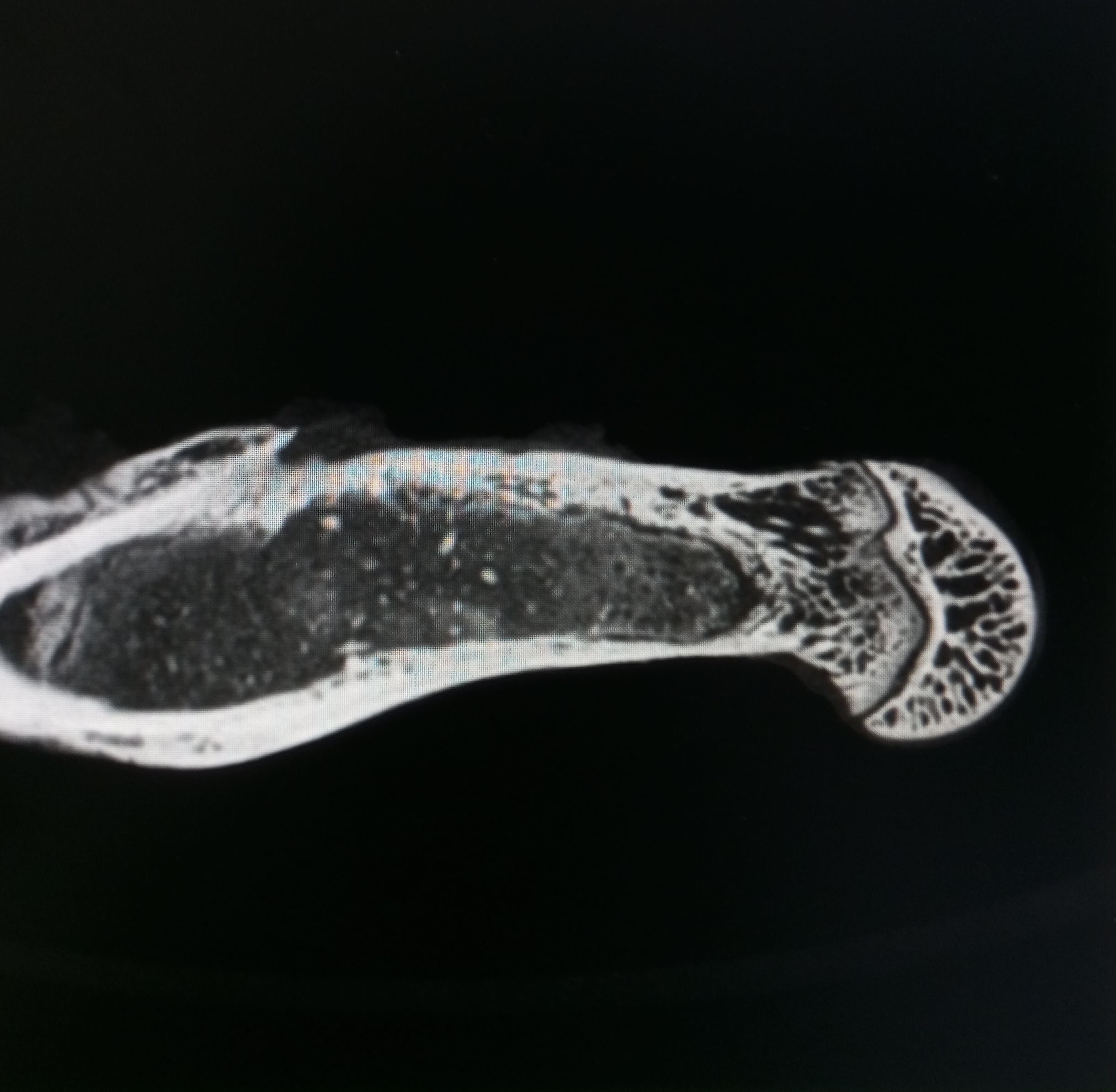

Supplement: Supplementary file 1 [file bioengineering-12-00599-s001.zip › supplementary materials/micro-CT images/Mg@Ca/4 weeks 2.jpg]

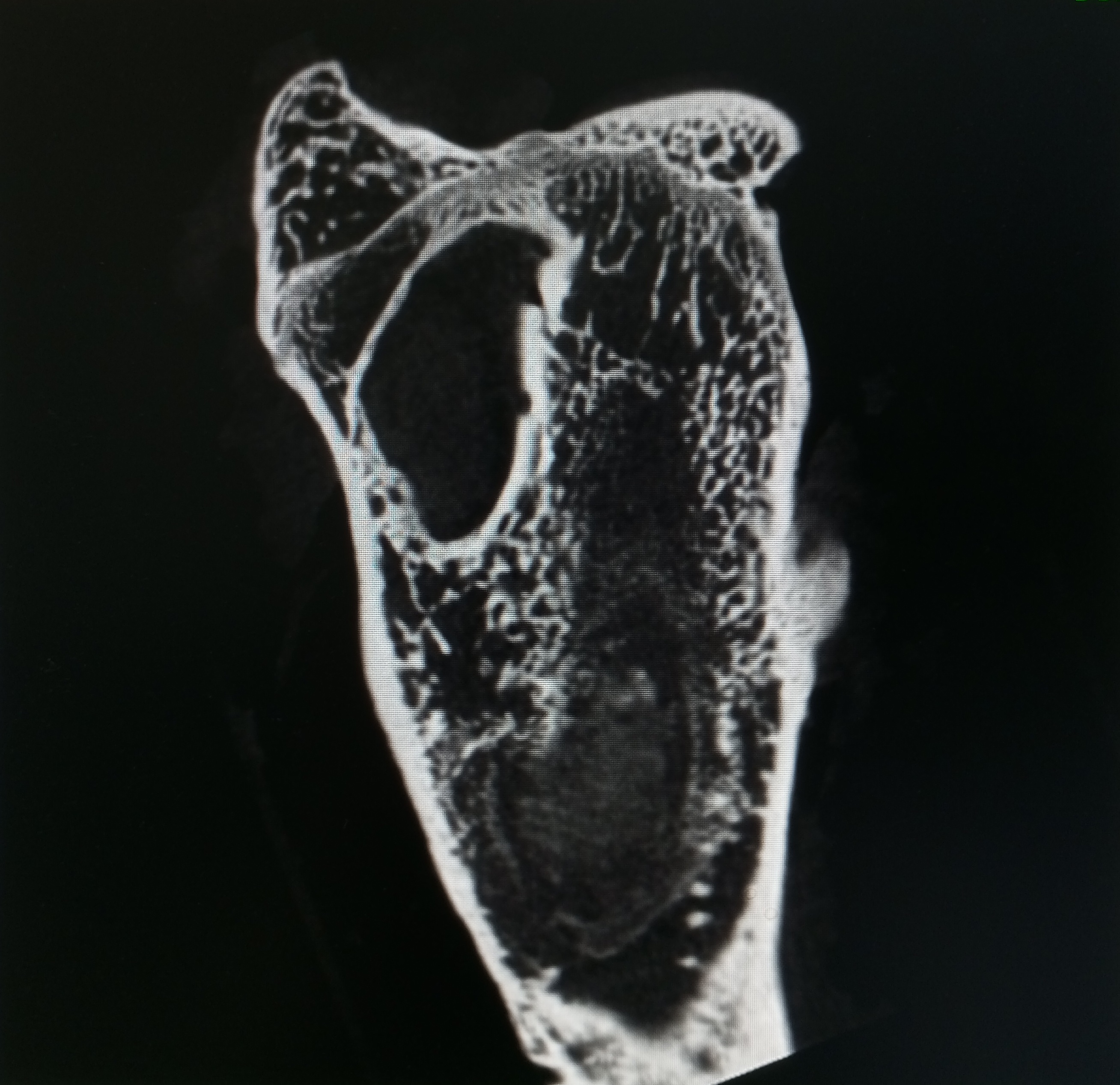

Supplement: Supplementary file 1 [file bioengineering-12-00599-s001.zip › supplementary materials/micro-CT images/Mg@Ca/8 weeks 1.jpg]

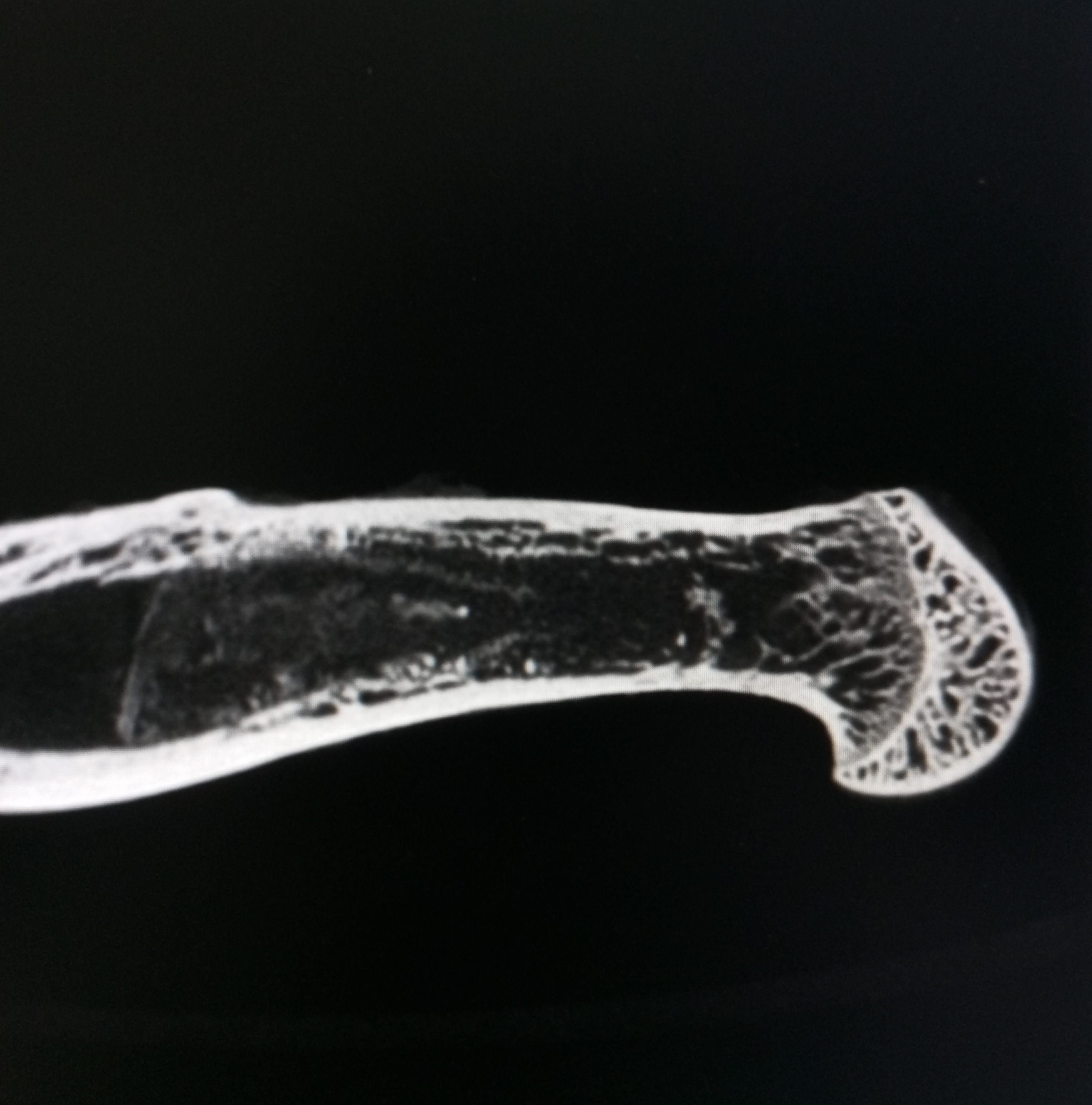

Supplement: Supplementary file 1 [file bioengineering-12-00599-s001.zip › supplementary materials/micro-CT images/Mg@Ca/8 weeks 2.jpg]

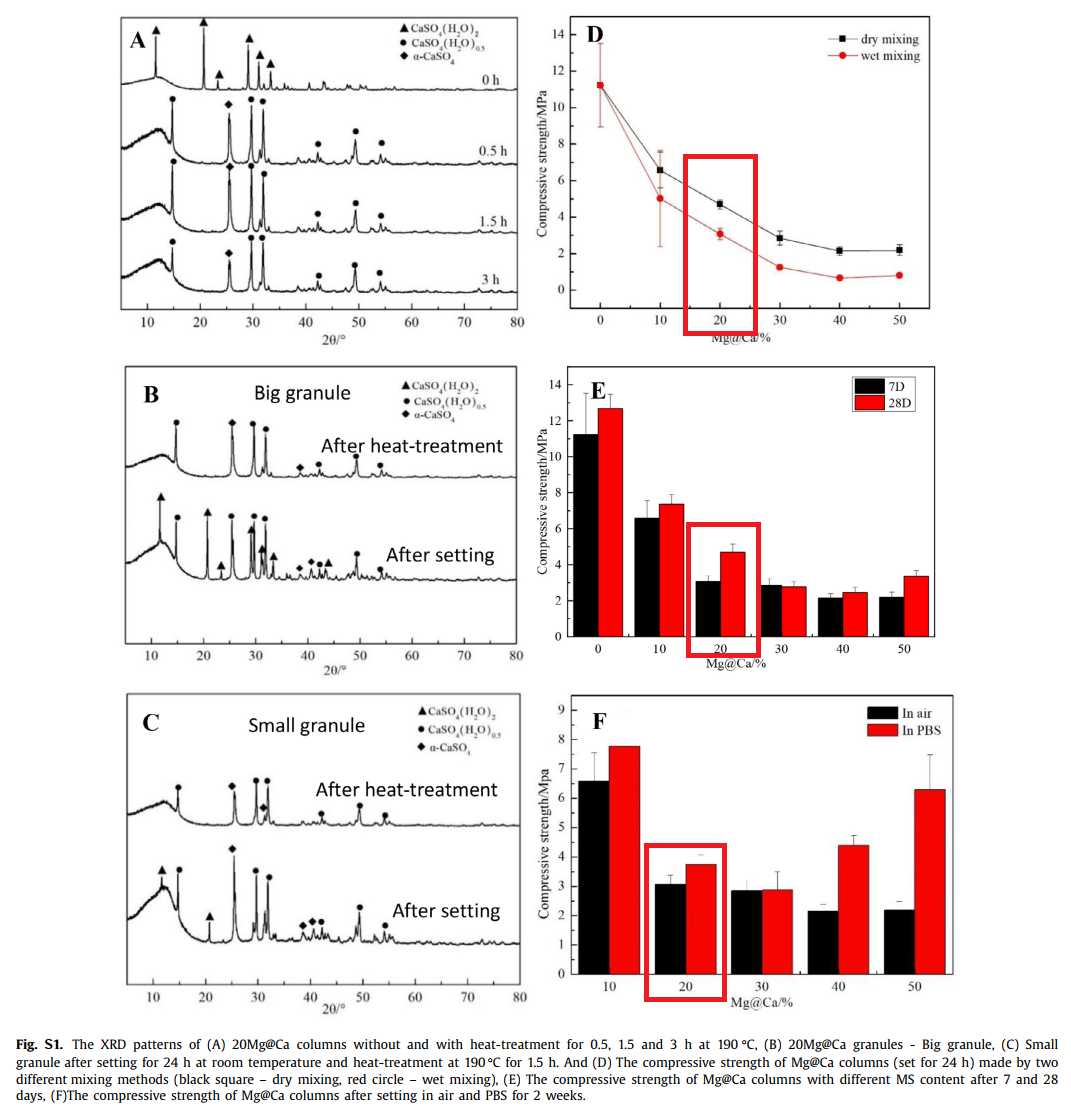

Supplement: Supplementary file 1 [file bioengineering-12-00599-s001.zip › supplementary materials/supplementary figures/Figure S1.png]

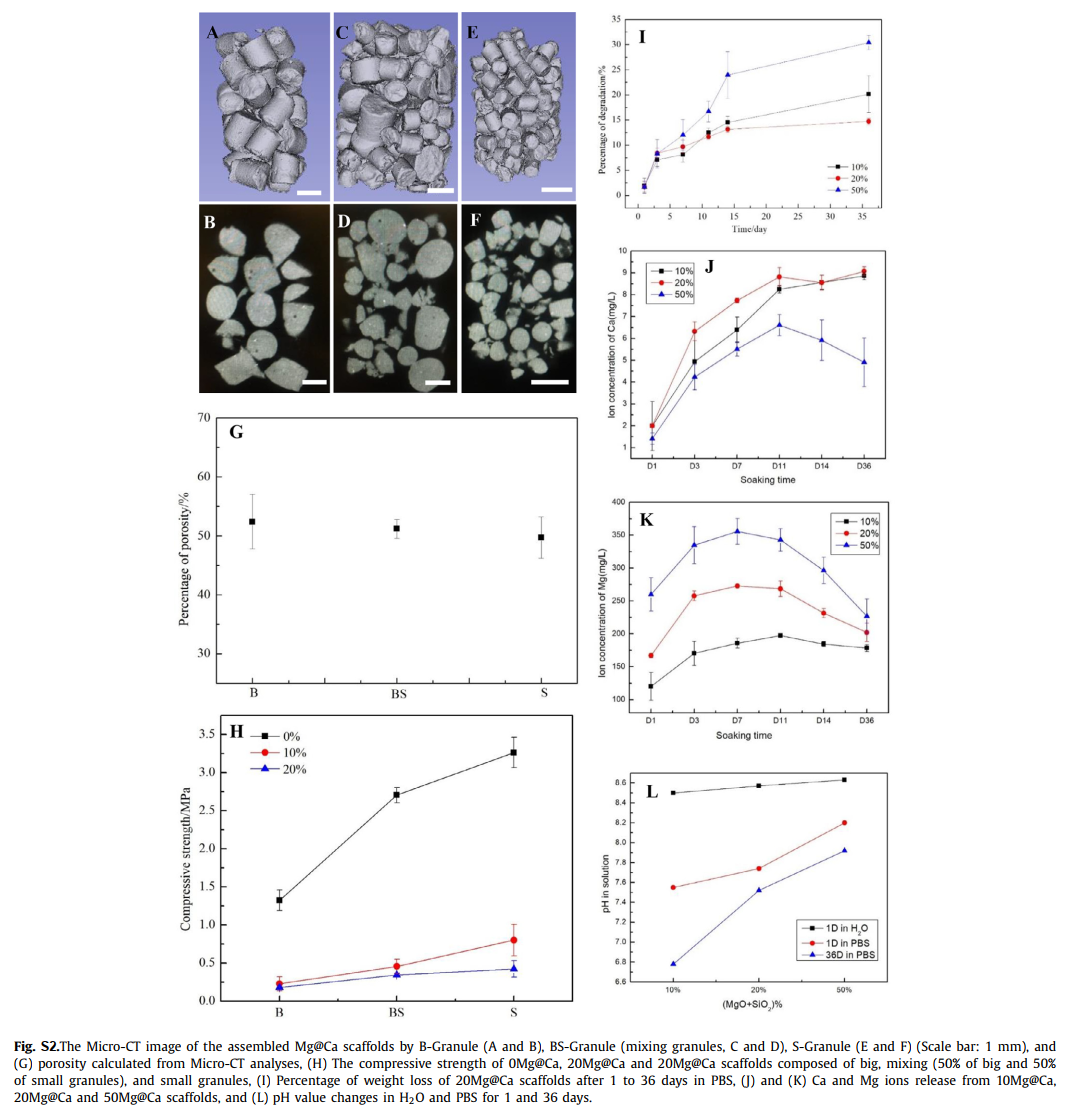

Supplement: Supplementary file 1 [file bioengineering-12-00599-s001.zip › supplementary materials/supplementary figures/Figure S2.png]
